# Structure of the PCBP2/stem–loop IV complex underlying translation initiation mediated by the poliovirus type I IRES

**DOI:** 10.1093/nar/gkaa519

**Published:** 2020-06-18

**Authors:** Simone A Beckham, Mehdi Y Matak, Matthew J Belousoff, Hariprasad Venugopal, Neelam Shah, Naveen Vankadari, Hans Elmlund, Joseph H C Nguyen, Bert L Semler, Matthew C J Wilce, Jacqueline A Wilce

**Affiliations:** Monash Biomedicine Discovery Institute and Department of Biochemistry and Molecular Biology, Monash University, Victoria 3800, Australia; Monash Biomedicine Discovery Institute and Department of Biochemistry and Molecular Biology, Monash University, Victoria 3800, Australia; Monash Biomedicine Discovery Institute and Department of Biochemistry and Molecular Biology, Monash University, Victoria 3800, Australia; The Ramaciotti Centre for Cryo-Electron Microscopy, Monash University, Victoria 3800, Australia; Monash Biomedicine Discovery Institute and Department of Biochemistry and Molecular Biology, Monash University, Victoria 3800, Australia; Monash Biomedicine Discovery Institute and Department of Biochemistry and Molecular Biology, Monash University, Victoria 3800, Australia; Monash Biomedicine Discovery Institute and Department of Biochemistry and Molecular Biology, Monash University, Victoria 3800, Australia; Department of Microbiology and Molecular Genetics, School of Medicine, University of California, Irvine, CA 92697-4025, USA; Department of Microbiology and Molecular Genetics, School of Medicine, University of California, Irvine, CA 92697-4025, USA; Monash Biomedicine Discovery Institute and Department of Biochemistry and Molecular Biology, Monash University, Victoria 3800, Australia; Monash Biomedicine Discovery Institute and Department of Biochemistry and Molecular Biology, Monash University, Victoria 3800, Australia

## Abstract

The poliovirus type I IRES is able to recruit ribosomal machinery only in the presence of host factor PCBP2 that binds to stem–loop IV of the IRES. When PCBP2 is cleaved in its linker region by viral proteinase 3CD, translation initiation ceases allowing the next stage of replication to commence. Here, we investigate the interaction of PCBP2 with the apical region of stem–loop IV (SLIVm) of poliovirus RNA in its full-length and truncated form. CryoEM structure reconstruction of the full-length PCBP2 in complex with SLIVm solved to 6.1 Å resolution reveals a compact globular complex of PCBP2 interacting with the cruciform RNA via KH domains and featuring a prominent GNRA tetraloop. SEC-SAXS, SHAPE and hydroxyl-radical cleavage establish that PCBP2 stabilizes the SLIVm structure, but upon cleavage in the linker domain the complex becomes more flexible and base accessible. Limited proteolysis and REMSA demonstrate the accessibility of the linker region in the PCBP2/SLIVm complex and consequent loss of affinity of PCBP2 for the SLIVm upon cleavage. Together this study sheds light on the structural features of the PCBP2/SLIV complex vital for ribosomal docking, and the way in which this key functional interaction is regulated following translation of the poliovirus genome.

## INTRODUCTION

Internal ribosomal entry sites (IRESs) are *cis*-acting RNA structures that facilitate non-canonical RNA translation initiation. They were originally discovered in members of the picornavirus family that possess a directly translated positive-strand RNA genome (1–3). Through the formation of specific RNA secondary structures, these RNA elements are able to recruit the host cell ribosomal machinery without the need for a 5′-cap as characteristic of host mRNAs. They have been classified into classes of IRES that vary in complexity - from the simplest Types III and IV (∼300-nt and 200-nt length respectively) that can directly recruit the 40S ribosomal subunit, to the more complex Types I and II (of ∼450-nt in length) that must be preloaded with host protein eukaryotic initiation factors (eIFs) and IRES-transacting factors (ITAFs) for ribosome recruitment. There are also IRES types that are unassigned to a specific structural category (4–6). After docking, the 40S ribosomal subunit scans the RNA for the authentic AUG start codon, following which the complete 80S ribosome assembles and translation is commenced. Using this strategy, the virus hijacks the host cell machinery for the translation of its viral genome and subsequently is able to replicate its genomic RNA and assemble new virions. The structural basis for IRES-ribosome docking is of immense interest, for both insight into the biology of viral translation initiation and also for its potential as a non-mutable target for the development of antivirals (7).

**Figure 1.**
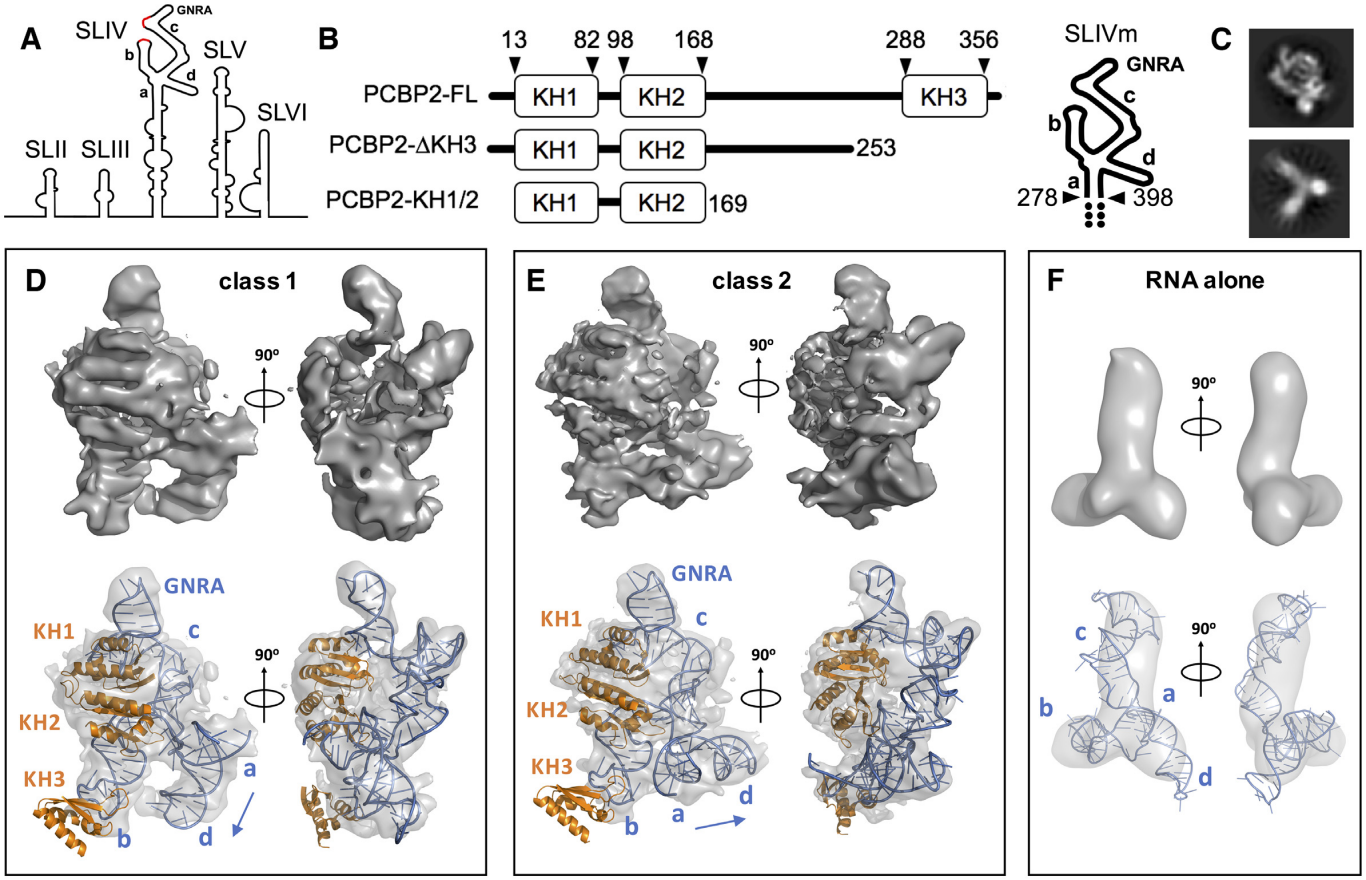
Cryo-electron microscopy analysis of PCBP2 interactions with SLIVm. (**A**) Schematic depicting the secondary structural arrangement of the poliovirus IRES. Highlighted are the C-rich regions (red) and GNRA tetraloop structure at the apex of SLIV. (**B**) Schematic representation of the protein and RNA constructs prepared for the current study. PCBP2 constructs included the full-length native sequence (PCBP2-FL), PCBP2 with KH3 removed from the 3CD cleavage site (PCBP2-ΔKH3) and a PCBP2 construct representing just the first two KH domains (PCBP2-KH1/2). The SLIVm RNA comprised bases 278–398 and three extra GC base pairs at the termini (depicted by dots). (**C**) Representative 2D classes for PCBP-FL/SLIVm (TOP) and SLIVm alone (bottom); (**D, E**) Top: Cryo-EM surfaces derived for class 1 and class 2 3D reconstructions of the PCBP2-FL/SLIVm complex. Bottom: constructed models of the PCBP2/SLIVm complex superposed with transparent cryo-EM surfaces to which the models were fitted. (**F**) Top: Cryo-EM surface derived for SLIVm alone from 3D reconstruction. Bottom: SLIVm model fitted to the cryo-EM surface, showing a similarity to the expected topology of the RNA.

The poliovirus IRES is one of the best characterized viral RNA elements and exemplifies the type I IRES of the picornavirus family that is also found in coxsackievirus B3 (CVB3), enterovirus 71 (EV71) and human rhinovirus 2 (HRV2) (8–10). It consists of five stem-loop (SL) structures (SLII to SLVI) spanning ∼450 nt near the 5′ end of its genomic RNA that provide binding sites for eIFs as well as several ITAFs required for docking of the 43S preinitiation complex (11,12) (Figure 1A). The two central stem-loops, SLIV and SLV, constitute the most important regions of the Type I IRES (13). SLV is bound by eIF4A and eIF4G that facilitate direct interactions with eIF3, a major component of the 43S preinitiation complex (14). SLIV is bound by ITAFs, including poly(C)-binding protein 2 (PCBP2) that is essential for translation of the viral genome (15,16) and SRp20 that augments translation (17). The other stem-loops serve as binding sites for additional ITAFs, including the La protein (18) and polypyrimidine tract-binding protein (PTB) (19). Each of these ITAFs has been shown to enhance the efficiency of poliovirus RNA translation. Of these, however, it is the PCBP2 interaction with SLIV that is most vital for effective translation (20). In the absence of PCBP2, poliovirus translation is severely impaired (21).

The essential nature of PCBP2 for poliovirus translation has led to its identification as a key regulator of viral genome translation. When PCBP2 is bound to SLIV of the poliovirus IRES, production of the viral proteins occurs as the first stage of viral replication. This includes the production of the poliovirus 3CD proteinase that, in addition to processing the viral encoded polyprotein, targets a number of cellular proteins for proteolytic cleavage, including PCBP2 (22). Upon translation of sufficient levels of viral proteins (including the 3CD proteinase), PCBP2 is proteolytically cleaved, resulting in a protein unable to mediate translation initiation (23,24). With the cessation of translation, viral genome replication that had been obstructed by ribosomes traversing the template in a 5′ → 3′ direction is no longer inhibited and is able to commence from the 3′ end of the RNA template as the next step toward the assembly of progeny virions (25). The PCBP2/SLIV complex is thus not only a key feature of the functional IRES RNP complex but also a molecular switch that regulates the transition from viral genome translation to the next stages of virion production.

Poly(C)-binding proteins (PCBPs) are ubiquitous ssRNA binding proteins that specifically interact with cytosine-rich tracts via their hnRNP K homology (KH) domains (26–28). The family includes PCBP1-4 and hnRNP K that all share a common architecture comprising a pair of N-terminal KH domains (KH1 and KH2) separated by a linker region of variable length from the C-terminal KH domain (KH3). Interactions with target RNA occur through each of the three KH domains that individually interact with approximately micromolar affinity, but together form a strong nanomolar binding affinity interaction with target RNAs (29,30). PCBP KH domains are ∼70 residue βααββα domains that forms a cleft between α1/α2 and β2/β3 that can accommodate four nucleotide bases (31). KH1 and KH3 have been structurally characterized in the presence of oligonucleotides (30,32,33). Specific hydrogen bond and electrostatic interactions underlie their preferential interactions with cytosine residues. Although the role of KH2 is less well understood, it is capable of binding to C-rich RNAs (34) and its mutation is disruptive to RNA binding (35). However, its individual contribution to RNA binding has not been quantitated, likely due to difficulty in the production of the isolated domain. KH2 forms a back-to-back dimer with KH1 via hydrophobic interactions and is thought to exist in this arrangement when interacting with RNA (34).

PCBP proteins bind to many target mRNAs (36) and serve a number of roles in the host cell, depending on the ternary complex in which they are involved. In the nucleus, PCBPs have been shown to impact on splicing and polyadenylation (37,38). In the cytosol, PCBPs impact mRNA stabilization (39–42) or translation repression (43–45), or they can enhance translation (46,47). The common feature of their function appears to be the stabilization of RNA secondary structures, such as the ‘alpha-complex’ formed at a specific C-rich region of the 3′-UTR of alpha-globin mRNA, causing its accumulation during terminal erythroid differentiation (48,49). This RNA-stabilizing function may also underlie the adoption of PCBP2 by viral RNA. PCBPs have been found to contribute to the overall stability of the poliovirus genome by binding to the 5′-SLI cloverleaf structure that is important for RNA replication (50). This is an RNA structure that is distinct from the poliovirus IRES (35). It functions in viral RNA replication and acts as a promoter for the viral RNA-dependent RNA polymerase, possibly by forming a protein-mediated bridge with the 3′ poly(A) tract (51,52). It is likewise possible that the interaction of PCBP2 with poliovirus SLIV serves to stabilize the SLIV structure within the IRES.

Due to its importance, the PCBP2/SLIV interaction has been the subject of detailed investigation. Poliovirus SLIV has been established as forming a 207-nt cruciform structure with loops emanating from the stem (designated stem **a** and loops **b**, **c** and **d** in the current study; Figure 1A) (11,53). Footprinting and mutational analysis have established that PCBP2 makes specific interactions with poliovirus SLIV via binding to C-rich regions in loops **b** and **c** as well as a ‘bulge’ in loop **c** (54) as it does in comparable regions of coxsackievirus B3 SLIV (55). As well as interactions via the KH domains, it has also been shown that the linker region of PCBP2 contributes to SLIV binding (55). More recently, PCBP2 sites have been localized to specific regions of poliovirus SLIV using hydroxyl radical cleavage directed by the strategic placement of cysteine residues within the PCBP2 structure (20,56). This has revealed the location of PCBP2 KH1 specifically at loop **c** and the ‘bulge’ region, whereas KH2 and KH3 appear predominantly close to loop **b**, but also in the vicinity of loop **d**. These finding have led to the proposal that PCBP2 binds to SLIV in such a way as to promote the function of a conserved GNRA (N = any nucleotide, R = A or G) tetraloop that is positioned at the apex of SLIV loop **c**, promoting ribosomal docking (56).

Although structural information is available for individual PCBP2 KH domains and short regions of oligonucleotides, no structural information is yet available for the PCBP2/SLIV complex that could advance our understanding of this critical feature of the poliovirus IRES. The current study was therefore undertaken to directly determine the three-dimensional arrangement of PCBP2 in complex with the target region of poliovirus SLIV. We designed a modified version of SLIV that included only the C-rich apical region (nucleotides 278–398) of SLIV, stabilized with three extra GC pairs at the stem region (henceforth referred to as SLIVm; Figure 1B). The SLIVm RNA formed a tight 1:1 complex with PCBP2 and was analysed using cryo-electron microscopy, despite being only 79 kDa in size. We also subjected the complex to SEC-SAXS (size exclusion chromatography-coupled small angle X-ray scattering) as a comparative technique and to obtain the flexibility information that can be derived from SAXS data. This method allowed us to directly compare the structure and flexibility of SLIVm before and after PCBP2 binding, and also determine the effect of PCBP2 cleavage by studying a cleaved form of PCBP2 (PCBP2-ΔKH3). Together with SHAPE (Selective 2′-hydroxyl acylation analysed by primer extension), hydroxyl radical footprinting, limited proteolysis and binding studies, we present the most advanced model of the PCBP2/SLIV complex to date that sheds light on poliovirus IRES function and regulation.

## MATERIALS AND METHODS

### Protein production

PCBP2-FL (full-length) and PCBP2-ΔKH3 (residues 1–253) were expressed using plasmids pET22b-PCBP2-FL and pET28a-PCBP2-ΔKH3 in *E. coli* Rosetta 2 (Merck) by incubation at 37°C to an OD_600_ of 0.6. Protein production was induced with 1 mM IPTG for 3.5 h at 25°C. Protein from disrupted cells was bound to Ni-NTA agarose (QIAGEN), washed with buffer containing 20 mM imidazole followed by a 2 M NaCl salt wash and eluted with 60 mM imidazole. After dialysis in a low salt buffer the protein was purified by size-exclusion chromatography using a Superdex 75 10/300 column (GE LifeSciences) followed by anion exchange using a MonoQ 5/5 column. For the production of PCBP2-KH1/2 (residues 13–169), pET15b-PCBP2-KH1/2 was generated, by cloning into the *Nde*I and *Xho*I sites of pET15b. The plasmid was transformed into *E. coli* Rosetta 2 (Merck), and expression, refolding and purification was performed according to Du *et al.* (34). Fractions were assessed by SDS-PAGE, pooled and buffer exchanged into 5 mM Tris, 5 mM KCl, 0.5 mM DTT, 5% glycerol, pH 8.0 and stored at –80°C.

### Preparation of DNA templates

#### SLIVm RNA

The DNA template for SLIVm encoding nucleotides 278–398 of the poliovirus RNA, was produced by PCR using the plasmid pT220-460 (provided by Dildine and Semler) as a template, with a forward oligonucleotide directed against nucleotides 278–290 and encoding the T7 RNA polymerase promotor site at the 5′-terminus (5′TAATACGACTCACTATAGGGCGCGTTGCGCTCAG-3′), and a reverse oligonucleotide designed against nucleotides 380–398 (5′-GGCGCGTCCCATGGCGTTAG-3′, GeneWorks, Australia), both oligonucleotides were designed to encode three extra GC pairs beyond the native sequence to stabilize the termini of the RNA. The PCR was performed with *Pfu* DNA polymerase (Promega) for 30 cycles of 94°C/30 s, 42°C/20 s, 72°C/30 s.

#### SLIVm-SHAPE RNA

The RNA design for SHAPE (Selective 2′-hydroxyl acylation analysed by primer extension) analysis was based on Wilkinson *et al.* (57). A 14-mer hairpin linker was included at the 5′-terminus of SLIVm and a hairpin linker and reverse transcriptase primer binding site at the 3′-terminus. A plasmid encoding this fragment was generated by GeneArt Gene Synthesis (Thermo Fisher Scientific). The DNA template was amplified by PCR using this plasmid as a template with sequence specific oligonucleotides (5′-GAATTCTAATACGACTCACTATAGGGCCTTCGGG-CCAGGGCG-3′, (Micromon Genomics, Monash University, Australia) and a reverse oligonucleotide designed against the reverse transcriptase primer binding site located at the 3′ end of the template (5′-GAACCGGACCGAAGCCCG-3′ GeneWorks, Australia), using *Pfu* DNA polymerase (Promega), for 30 cycles of 94°C/30 s, 42°C/20 s, 72°C/30 s.

### Preparation of RNA

RNA from the PCR amplified DNA template was transcribed using the T7 RiboMAX Express Large Scale RNA Production System (Promega), extracted using phenol/chloroform and precipitated using 0.3 M sodium acetate in 100% ethanol, washed with 70% ethanol, resuspended in TE (10 mM Tris pH 8.0, 1 mM EDTA) and buffer exchanged to remove unincorporated rNTPs using an Amicon Ultra centrifugal concentrator (Merck). To ensure correct folding, RNA was annealed immediately before use in experiments.

### Protein–RNA complex formation and verification using SEC-MALS

The quality of the protein, RNA and complexes were assessed using size-exclusion chromatography-multi-angle light scattering (SEC-MALS). For complex formation, 50 μg of RNA was diluted to 30 μM in TE, and annealed for 5 min at each of 68°C, 37°C, 21°C. A 1.5 molar excess of protein was incubated in RNA binding buffer (5 mM HEPES-KOH, 25 mM KCl, 2 mM MgCl_2_, 2 mM DTT, 4% glycerol, 0.1 mM EDTA, pH 7.5) at 30°C for 10 min at ∼3.5 μM and added to the annealed RNA, bringing the concentration of the complex to ∼3 μM. The sample was incubated for a further 10 min at 30°C, then concentrated to 110 μl in a centrifugal concentrator at 21°C. 100 μl samples were injected onto a Superdex 200 10/300 column or S75 10/300 column (GE LifeSciences) in RNA binding buffer at a flow rate of 0.4 ml/min. Elution profiles and molecular masses were estimated using an SEC-MALS instrument consisting of a Shimadzu Prominence UFLC, Shimadzu SPD-20A UV–Vis for UV data collection at 280 and 260 nm, a DAWN HELEO-II and an Optilab T-rEX (Wyatt Technology). Scattering and refractive index data were collected using ASTRA6 (Wyatt Technology).

### CryoEM data collection

PCBP2-FL/SLIVm complex was collected from SEC, concentrated to 2.6 μM and detergent n-octyl glucoside added to 145 nM. Prior to sample application, Quantifoil R1.2/1.3 holey carbon grids (Quantifoil GmbH, Großlöbichau, Germany) were glow discharged in the presence of 20 μl 99% *N*-amylamine (Sigma-Aldrich). A frozen hydrated specimen was prepared by applying 4 μl of sample on the grid in a Vitrobot Mark IV (FEI, Hillsboro, OR, USA) maintained at 4°C and 100% humidity. The sample was blotted with a blot force of +3 to +5 for 2.5 s and was plunge frozen into liquid ethane after a drain time of 1 s. Grids were transferred under liquid nitrogen to a Titan Krios transmission electron microscope (FEI, Hillsboro, OR, USA) operated at 300 kV, equipped with a K2 Summit™ direct detector (Gatan), GIF- Quantum energy filter (Gatan) and a Volta phase plate. Zero loss imaging was performed in nanoprobe EFTEM mode with the slit width set to 20 eV. C2 condenser aperture size of 50 μm and Volta phase plate was used in the objective plane. Movies were recorded at a nominal magnification of 130k corresponding to a calibrated magnified pixel size of 1.06 Å, dose rate of 4e/pixel/second, exposure time of 10 s, and a total dose of 40 e Å^−2^ which was fractionated into 40 subframes. Data collection was automated using SerialEM software package (58). Volta phase plate position was advanced every hour to account for phase shift saturation and autofocus was set to yield ∼0.5 μm defocus. A second data set was also collected with the stage tilted at an angle of 40° with the same microscope parameters.

### CryoEM image processing and 3D reconstruction

Dose fractionated movies were corrected for beam induced motion and electron dose by the MotionCor2 software package (59). A non-dose-weighted sum of each movie stack was then used for contrast transfer function (CTF) estimation and phase shift by CTFFIND (60). The micrographs that were collected while the stage was tilted had their CTF estimated on a non-dose weighted average on a 512 pixel box that was centred on the tilt axis using the gCTF software package (61). Particles recognized as PCBP2-FL/SLIVm were selected from 1377 un-tilted micrographs in a semi-automated fashion using both Gautomatch (developed by Kai Zhang at MRC Laboratory of Molecular Biology) and Relion 2.1 (62,63) software packages. 744 895 particles were then subjected to multiple rounds reference free 2D classification and class selection in both the Relion 2.1 and SIMPLE (64) software packages until classes corresponding to what appeared to be the complex of interest were predominant. This corresponded to a set of 265 865 particles. An initial 3D model was generated using the SIMPLE software package with using the Initial Model from Class Averages subroutine.

Particles were also selected from 100 tilted micrographs in a semi-automated fashion using Relion 2.1. Particles within a 1000-pixel width rectangle along the axis of tilt were then extracted which corresponded to 74 535 particles where then subjected to multiple rounds of reference free 2D classifications and selection. The final subset corresponded to 11 640 particles which were then combined with the un-tilted data and the combined particle set was subjected to 3D classification in Relion 2.1, where two distinct 3D conformations were apparent corresponding to 88 988 and 75 014 particles respectively. These two classes where then further refined and post-processed in Relion 2.1. They refined to a global resolution of 6.1 and 6.4 Å according to the gold standard Fourier shell correlation (FSC) criterion of 0.143 (Supplementary Figure S2).

A significant number of particles recognized as uncomplexed SLIVm RNA were observed on the micrographs. The corrected movie stacks (described above) were imported into CRYOSPARC (65). Particle picking (blob picker) identified 882 404 particles. Cycles of 2D classification and selection were performed to select for RNA particles. Homogeneous refinement resolved one class which included 12 137 particles to a global resolution of 9.6 Å according to the gold standard Fourier shell correlation (FSC) criterion of 0.143 (Supplementary Figure S2).

### Small angle X-ray scattering data collection

Samples were prepared as described for SEC-MALS and concentrated to ∼100 μM in a centrifugal concentrator. Using a Shimadzu Prominence UFLC, 100 μl samples were injected onto a Superdex 200 10/300 or S75 10/300 column (GE LifeSciences) in RNA binding buffer at a flow rate of 0.4 ml/min in direct connection with the SAXS/WAXS beamline at the Australian Synchrotron. Coflow, a sheath flow capillary system (66), was employed using the same bottle of RNA binding buffer for the sheath solution as well as the chromatography, to ensure exact buffer matching. Scattering data were collected continuously during SEC elution on a 1M Pilatus detector at 15°C and at a distance of 2.68 m for 5 s exposures at 0.05 s intervals. The wavelength was 12 keV and beam dimensions of 120 × 120 μm.

### Processing of SAXS data

The Australian Synchrotron SAXS/WAXS beamline specific software, SCATTERBRAIN, was utilized for SAXS data reduction (including radial integration of the SAXS images and conversion to 3 column dat files). SEC-SAXS data were converted to the time domain with the HPLC SAS module of the program *Ultrascan Solution Modeler* (*US-SOMO*) (67). Typically, 100 frames were selected after the flow-through had cleared the column for background subtraction. The background frames were averaged with DATAVER (68) and then subtracted from all of the remaining frames using DATOP (68). Due to the presence of overlapping peaks in some of the SEC profiles the HPLC SAS module of US-SOMO was employed to deconvolute the data (67,69). The top 5% of frames in each deconvoluted peak were averaged and stored as dat files. ATSAS programs were used for further analyses including AUTORG for Guinier calculation (70), DATGNOM for calculation of pairwise distribution functions (70), and dimensionless Kratky plots were calculated using the DATGNOM fitted scattering curves with scripts written by MCJW. DAMMIF (71) was used to calculate *ab initio* dummy atom models from the scattering data. Fifty SLIVm alone models were generated and DAMCLUST (72) used to group them from which the largest cluster was selected. In the case of PCBP2-FL/SLIVm, 15 models were generated and were superposed, merged and filtered using the program DAMAVER (73).

### Molecular modelling of the PCBP2/SLIVm complex

A complete structure of PCBP2 was modelled using PHYRE (74) with the ‘intensive’ option, coordinates for KH1 and KH2 domains of PCBP2 were obtained from PDBID 2JZX (34), the third KH domain from PDBID 2P2R (32). The missing N-terminal and C-terminal residues and the linker between KH2 and KH3 were modelled *ab initio*. Coordinates for SLIVm were calculated using RNAexplorer (75) based upon the secondary structure predicted by MFOLD (http://unafold.rna.albany.edu/?q=mfold). The MMTSB Toolbox (76) was used to prepare coordinates for CHARMM version 35b5 (77). The coordinates of SLIVm and PCBP2-FL were combined to make a complex using CHARMM with NOE constraints to position KH1 and KH3 on the appropriate C-rich regions of SLIVm. The coordinates of SLIVm were fixed except for the C-rich regions during this procedure. The NOE constraints were defined based upon the hydrogen bonding between the protein and RNA within crystal structures PDBID 1AXY for KH1 and PDBID 2P2R for KH3 as defined by PISA (https://www.ebi.ac.uk/pdbe/pisa/).

### Molecular dynamic with flexible fitting

The coordinates of the poliovirus SLIVm/PCBP2 complex were fitted into the cryo-EM maps using the plugin MDFF as implemented in NAMD2 and outlined in the MDFF NAMD tutorial (http://www.ks.uiuc.edu/Training/Tutorials/science/mdff/tutorial_mdff-html/tutorial_mdff.html). Prior this the complex model was roughly fitted into the cryoEM map using COLORES from the Situs package (https://situs.biomachina.org/). MDFF utilized the generalized Born implicit solvent model, and, domain restraints were applied using targeted MD (TMD) throughout and included the first two domains of PCBP2 (KH1 and KH2) and the third KH domain, RNA domains were defined in a manner as described above in the rigid body molecular dynamics of RNA alone. Protein termini and domain linkers were not restrained. The elastic constant of the TMD forces was set to 1000 kcal/mol/Å^2^ and the initial and final TMD rmsds were maintained at 0.1 Å. Interactive molecular dynamics was used to guide the coordinates into the cryoEM maps. The GSCALE parameter was changed from 0.1 stepped through to a value of 10 during the MDFF. NAMD ExtraBonds were exploited to hold the KH domains bound to their cognate RNA binding sites during the MD. A total of 50 ns of MD was performed during the IMD and further 50 ns was performed to allow for equilibration. KH3 of PCBP2 and the linker between KH2 and KH3 were excluded from fitting into the cryoEM map.

### Flexible ensemble search

CHARMM was employed to carry out the procedures as described by Pelikan (78) using scripts written by MCJW to generate an ensemble of PCBP2/SLIVm structures that best fit the SAXS scattering data. The PCBP2/SLIVm model that had been fitted in the Class 1 cryoEM map was used subject to rigid body molecular dynamics. In summary, the coordinates underwent energy minimization followed by rigid body dynamics with *R*_G_ constraints to allow a wide yet restrained amount of conformational space to be explored. The coordinates of KH3 and SLIVm residues 275–312 and 374–401 were kept fixed, while KH1 and KH2 and SLIVm residues 323–361 were allowed to move as a rigid body. All other coordinates were free to move. Rigid body molecular dynamics trajectories of 1000 time points were generated for the following *R*_G_ restraints 32, 34, 36, 38, 40, 42 and 44 Å. SAXS curves were calculated for each point in the trajectories with FOXS (79) and the program Minimal Ensemble Search (MES) (78) provided the minimal set of coordinates that gave the best fit to the SAXS data. The single set of coordinates that best fit the scattering data was also identified.

### Selective 2′-hydroxyl acylation analysed by primer extension (SHAPE) and hydroxyl radical foot printing

SLIVm-SHAPE RNA and PCBP2-FL/SLIVm-SHAPE and PCBP2-ΔKH3/SLIVm-SHAPE complexes were prepared as described above in modified RNA binding buffer (5 mM HEPES–KOH, 25 mM KCl, 2 mM MgCl_2_ pH 7.5) and subjected to SEC-MALS. For SHAPE analysis, 8 μl of 60 mM NMIA in 100% DMSO was added to 60 pmol RNA or RNA-protein complexes to a final volume of 80 μl and was incubated for 37°C for 50 min. A no NMIA control containing 10% DMSO was also included and the reactions were quenched by the addition of glycerol to a final concentration of 15%. Hydroxyl radical cleavage was performed according to (80). Stocks of 7.5 mM Fe(II)/11.25 mM EDTA (pH 8.0), 0.3% H_2_O_2_ and 150 mM ascorbic acid (sodium salt) were freshly prepared. 60 pmol RNA or RNA–protein complex was prepared in 74 μl of modified RNA binding buffer with 2 μl of each of the Fe(II)/EDTA, H_2_O_2_ and ascorbic acid solutions, or 6 μl of nuclease-free water as a no reagent control. The samples were incubated at 37°C for 5 min and the reactions quenched by the addition of glycerol to 15%.

Both the SHAPE and hydroxyl radical cleavage samples were digested with 10 μg/ml proteinase K at 37°C for 10 min. The modified/cleaved RNA was extracted using phenol/chloroform, precipitated and resuspended in TE. 6 pmol of RNA was annealed with a 5′-6-FAM-labeled oligonucleotide (5′-6-FAM-GAACCGGACCGAAGCCCG-3′, Applied Biosystems) for 5 min at each of 65°C, 35°C and on ice, according to Wilkinson *et al.* (57) and incubated with SuperScript III Reverse Transcriptase (Invitrogen) for 1 h at 50°C. The RNA was digested with 0.2 M NaOH at 95°C for 5 min, then neutralized with 0.2 M HCl and placed on ice. The cDNA was then precipitated and washed. A ddCTP ladder was generated using a reverse transcriptase reaction containing non-treated RNA and a VIC-labelled oligonucleotide (5′-VIC-GAACCGGACCGAAGCCCG-3′, Applied Biosystems). The cDNA fragments were then precipitated. The samples and ladder were resuspended in Hi-Di formamide (Applied Biosystems) combined and submitted to the Monash Health Translation Precinct Medical Genomics Facility for fragment analysis on an Applied Biosystems 3130xl Genetic Analyser, using the G5 dye set. The SEC-MALS preparation of the samples and the SHAPE and Hydroxyl radical reactions were performed in duplicate using different batches of RNA.

### SHAPE and hydroxyl radical cleavage data processing

QuSHAPE (81) was used to generate normalized SHAPE and hydroxyl radical cleavage reactivity values. The fragment analysis capillary electrophoresis data for RNA treated with NMIA or the Fenton reaction reagent data was subtracted from their respective no reagent data. QuSHAPE was used to adjust for differences in mobility of the fluorophores used for the reactions and the ladder, and alignment of the ladder and base calling was performed. Default parameters were applied for Smoothing, Signal Decay Correction and Gaussian peak fitting. For SHAPE analysis, scaling was performed against the nucleotide sequence exhibiting the lowest reactivity values in the presence of the reagent, corresponding to nucleotides 278–395 of the poliovirus sequence. For the hydroxyl radical cleavage, scaling against the no-reagent data was performed against three nucleotides (nucleotide 286 at the 5′ end of the stem **a** structure and nucleotide 377 at the 5′ end of the stem of loop **d**). Default normalization was applied, which resulted in 1.65–1.68% outlier values.

### Limited proteolysis with trypsin

PCBP2 (80 μg) was prepared with and without an equimolar amount of annealed SLIVm and concentrated to a volume of 70 μl. 0.4 μg of sequencing grade modified trypsin (Promega) was added and the sample incubated at 30°C. 10 μl of sample was removed prior to trypsin addition and at each time point 0, 10, 30, 60, 90 and 120 min, and the digest stopped by the addition of SDS-PAGE loading dye and heating 95°C for 5 min followed by storage on ice. The samples were subjected to electrophoresis on a Mini-Protean TGX Precast gel (Bio-Rad) and visualized using Coomassie Blue. The cleavage products were separated by SDS-PAGE, transferred to PVDF and submitted to the Monash Biomedical Proteomics Facility for N-terminal sequencing by Edman degradation.

### RNA electrophoretic mobility shift assay

SLIVm RNA was biotinylated using the Pierce RNA 3′ End Biotinylation Kit (Thermo Fisher Scientific) according to the manufacturer's protocol. RNA mobility shift assays were performed as described previously (35,82). Purified PCBP2-FL or PCBP2-ΔKH3 were preincubated with 1 mg/ml tRNA (Roche) in binding buffer (5 mM HEPES-KOH pH 7.5, 25 mM KCl, 2.5 mM MgCl_2_, 3.8% glycerol, 20 mM DTT) for 10 min at 30°C. Biotinylated RNA probe and 8 U of RNAsin (Promega) were then added followed by incubation for 10 min at 30°C. The reaction mixture was further incubated with 0.5 mg/ml of bovine serum albumin (Promega) for 10 min at 30°C. Following incubation, 1.25 μl of 80% glycerol was added. The samples were separated by native TBE-PAGE containing 5% acrylamide at 4°C, electrophoretically transferred to a nylon membrane (Roche), UV-crosslinked to the membrane using a Stratalinker UV Crosslinker 1800 (Stratagene, USA). The biotinylated RNA on the membranes was visualized using a LightShift Chemiluminescent RNA EMSA kit (Thermo Fisher Scientific) on a Chemidoc Touch Imaging System (Bio-Rad) and quantified using ImageQuant TL 8.1 software (GE Healthcare). The fraction bound was calculated as the intensity of bound SLIVm RNA as a fraction of the total intensity of bound and free SLIVm RNA probe.

## RESULTS

### CryoEM structure of the PCBP2-FL/SLIVm complex

To obtain three-dimensional structural information using cryoEM for PCBP2 in complex with poliovirus SLIV, we prepared pure recombinant full-length PCBP2 (PCBP2-FL) and incubated it with a modified stem–loop RNA representing the apex of the poliovirus SLIV (nt 278–398), henceforth referred to as SLIVm. The T7 RNA polymerase generated 127-nt RNA included loops **b, c** and **d** previously established to be the target binding sites of PCBP2 KH domains (20,56) as well as three extra G-C base pairs designed to constrain the termini of this cruciform RNA (Figure 1B). The complex of SLIVm and PCBP2-FL was purified using size exclusion chromatography (SEC), verified as a 1:1 stoichiometric complex using multi-angle light scattering (MALS) determination of molecular mass (Supplementary Figure S1A; Table S1) and subjected to cryoEM structure determination.

Despite the relatively small size of these molecular components (PCBP2-FL = 39.6 kDa; SLIVm = 39.2 kDa), it was possible to resolve the PCBP2-FL/SLIVm complex particles within the thin ice sections of the holey carbon grids for 2D classification (Supplementary Figure S2). In addition, particles representing RNA alone were apparent. Representative 2D classes for the PCBP2/SLIVm complex and the RNA alone are shown in Figure 1C. Classes designated as PCBP2-FL/SLIVm complex were subjected to 3D classification resulting in two main structural classes. These were each reconstructed to 6.1 Å (class 1) and 6.4 Å (class 2) resolution, revealing globular well-defined structures consistent with PCBP2 interacting with the SLIVm loops through multiple KH domain interactions (Figure 1D&E). Protein and RNA models were built into the maps, boot-strapped by the high similarity of the map surface features to the expected structure of the PCBP2-KH1/KH2 domains that are known to exist as a back-to-back dimer (34). Rib-like stretches of density were matched with the positions of the KH1/KH2 α-helices. Since it is known that KH1 interacts with the C-rich sequence at the apical region of loop **c** of SLIV (20,56), the adjacent density was interpreted as loop **c** with the C-rich sequence positioned at the KH1 RNA binding site and the GNRA tetraloop protruding conspicuously outward. Interestingly, this protrusion is apparent, even in the 2D class images acquired, suggestive of a highly stabilized motif formed by the PCBP2-FL/SLIVm structure (Figure 1C).

For the first PCBP2/SLIVm structural class, the RNA stem of loop **c** was built into density arching between the KH1 binding site and the apparent four-way junction of the SLIVm structure. The junction was constructed as a type cH junction (83) with coaxial stacking of stem **a** and loop **d** and opposing directionality of loop **b** and loop **c** (similar to that observed in the HCV IRES (1KH6_4) despite these structures being unrelated) (Figure 1D). Loop **b** was built into the fourth branch of density with KH3 positioned at the apical C-rich binding site of loop **b**, beyond the well-defined density. Clear density was not apparent for the linker region between KH2 and KH3 though a small region of unfilled density is apparent adjacent to stem loop **c**. This 120 amino acid region is predicted to be unstructured, but is reported to contribute to SLIV binding (55). In the current structure, the KH3 and linker domain regions may possess a degree of flexibility and not provide clear density in the cryoEM reconstructed structures. The resulting model, built from a combination of known PCBP2 KH domain structures and *ab initio* shaping of the RNA from known base pairing topology represents the first experimentally-based 3D model of full-length PCBP2 bound to SLIV of the poliovirus IRES. The second structural class derived for the PCBP2-FL/SLIVm complex was similar in all respects to the first except for the density representing the position of the coaxially stacked stem **a** and loop **d** (Figure 1E). In this class, these features are effectively twisted at the four-way junction with respect to the rest of the structure by about 90 degrees. The existence of these two structural classes suggests that the PCBP2-FL/SLIVm construct possesses flexibility about its four-way junction.

The cryoEM data also included particles representing RNA alone that could be used to construct a structure for the SLIVm to 9.6 Å resolution (Figure 1F). SLIVm appeared with three main lobes and a hint of a fourth, consistent with the predicted cruciform structure. None of the lobes corresponded to the full-length expected for loop **c**, suggesting a bent conformation or a degree of flexibility in the absence of bound protein. There was no evidence of a well-defined GNRA tetraloop as apparent in the PCBP2-FL/SLIVm structures.

### Importance of the GNRA tetraloop

The above-described structures highlight the GNRA tetraloop as a prominent feature of the PCBP2/SLIVm complex and undiscernible in the SLIVm structure alone. To further interrogate the importance of the GNRA tetraloop in poliovirus translation and infectivity, we first analysed a mutated form of SLIV in which the GUGA tetraloop was substituted with the sequence GACG (refer to Supplementary Figure S7A). When the isolated version of this SLIV sequence (GNRA-Sub) was subjected to enzymatic RNA structure probing, no overall structural differences were observed compared to WT SLIV (*data not shown*). We then used this altered version of SLIV in RNA mobility shift assays with increasing amounts of recombinant PCBP2-FL. RNA transcripts harbouring the GNRA substitution (GNRA-Sub) were able to efficiently form a complex with recombinant PCBP2, albeit at reduced levels compared to the wild type SLIV RNA (Supplementary Figure S7B). DNA sequences corresponding to the GNRA-Sub version of SLIV were substituted into the full length cDNA corresponding to infectious poliovirus genomic RNA. *In vitro* transcripts derived from this mutated cDNA (along with those derived from wild type cDNAs) were tested in cell-free translation assays for their ability to direct viral protein synthesis. As shown by the SDS-PAGE analysis displayed in Supplementary Figure S7C, RNAs harbouring the GNRA-Sub mutation were unable to produce viral proteins in extracts derived from uninfected HeLa cells. This was in stark contrast to the wild type transcript, which produced nearly the full complement of viral proteins detected during a poliovirus infection (compare lanes 3, 4 to lanes 5, 6 and lanes 7, 8 to lanes 9, 10, respectively, in Supplementary Figure S7C). Not surprisingly, when these same transcripts were used in RNA transfection assays, no infectious virus could be recovered from the GNRA-Sub RNAs, while the wild type RNAs produced readily recovered infectious virus (*data not shown*). Taken together, these data reveal that although the GNRA tetraloop in SLIV is not essential for PCPB2 binding, it is crucial to the IRES function of poliovirus genomic RNA and, as a result, to virus infectivity.

### Conformational flexibility of PCBP2-FL/SLIVm and PCBP2-ΔKH3/SLIVm complexes assessed by SAXS

Concurrent with the cryoEM, we also obtained in-solution structural data using SEC-SAXS for PCBP2-FL, as well as truncated forms, PCBP2-ΔKH3 (residues 1-253) and PCBP2-KH1/KH2 (residues 13–169), before and after complex formation with SLIVm. PCBP2-ΔKH3 represents the inactivated form of PCBP2 after cleavage by 3CD (23). It contains only KH1/KH2 and 85 residues of the linker region and is no longer able to promote translation of the viral genome (23). The molecule is, however, still able to form a 1:1 complex with SLIVm, as verified by SEC-MALS determination of molecular mass (Supplementary Figure S1B; Table S1). We used SEC-SAXS in which SAXS data were acquired directly as the complex eluted from the size exclusion column. We applied Gaussian deconvolution to further ensure the separation of SAXS data arising from the PCBP2/SLIVm complex vs molecular species eluting slightly earlier or later from the column (Supplementary Figure S3).

**Figure 2.**
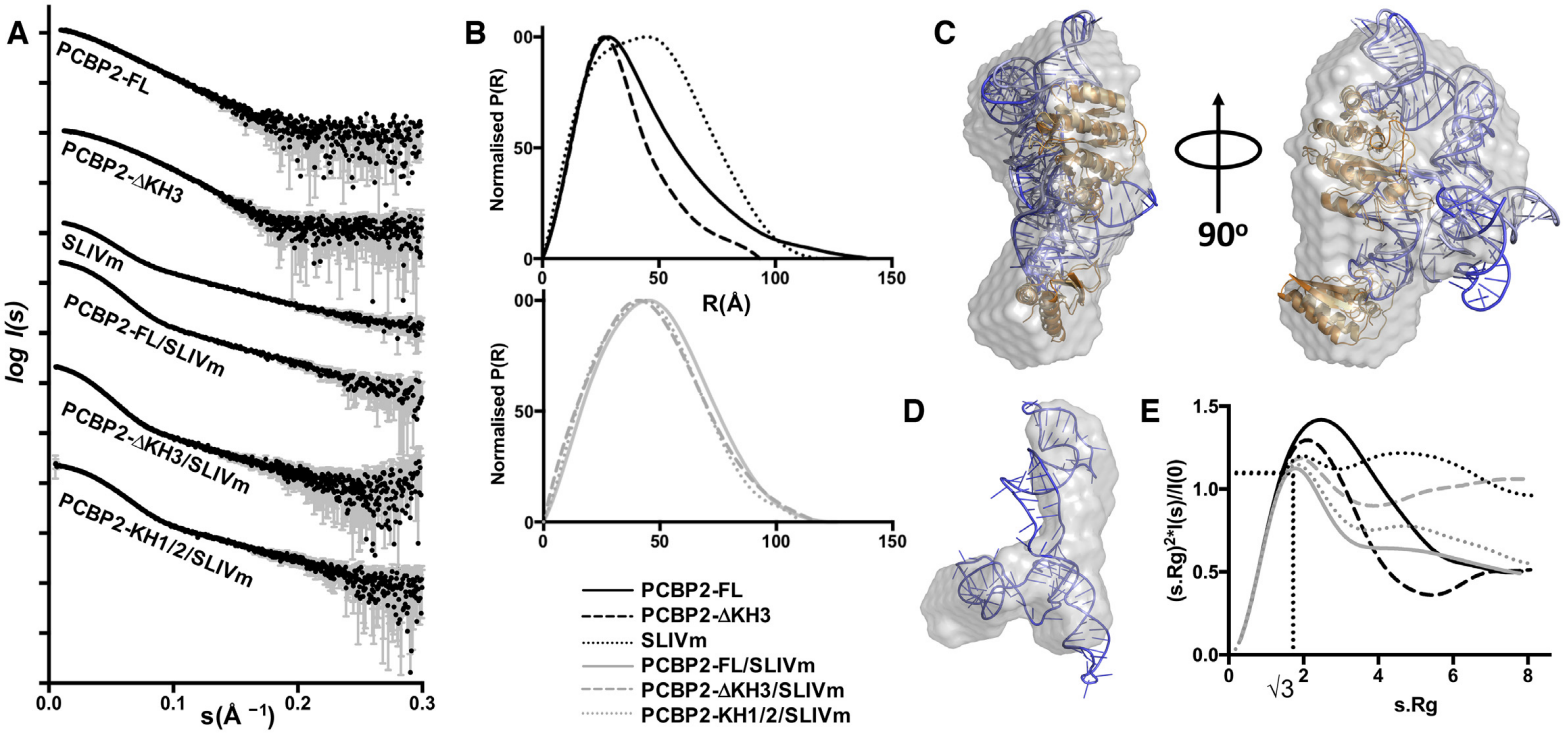
SAXS analysis of PCBP2 and SLIVm constructs before and after complex formation. (**A**) Experimental scattering profiles of PCBP2 constructs, SLIVm and PCBP2/SLIVm complexes, obtained by plotting the log of scattering intensity [log I(s)] as a function of forward scattering vector in the units of Å^−1^ and represented by black circles along with gray error bars showing the mean ± SD; (**B**) pairwise distribution [P(r)] profiles obtained from the scattering data by plotting P(r) as a function of r in the units of Å; (**C**) *ab initio* shape reconstruction derived for the PCBP2-FL/SLIVm complex using DAMMIF (NSD = 0.063) and represented as a grey envelope superposed with the cryoEM derived model; (**D**) *ab initio* shape reconstruction derived for SLIVm alone using DAMCLUST (NSD = 0.83) and represented as a grey envelope superposed with the cryoEM derived model. (**E**) normalized Kratky profiles derived from the scattering data.

Figure 2A shows the scattering curves obtained for PCBP2-FL and PCBP2-ΔKH3 constructs and SLIVm before and after complex formation. Converted into P(r) plots, it can be seen that while the maximal dimension is relatively long for apo-protein PCBP2-FL (*R*_max_ = 132 Å) a more compact globular structure is formed upon complex formation with the SLIVm (*R*_max_ = 115 Å) indicating a shorter maximal dimension and a profile shifted to the right indicating a larger average dimension (Figure 2B; Supplementary Table S2). The P(r) plot for the RNA alone shows a bimodal profile, consistent with an elongated feature being present. This feature shifts to the left upon complex formation with PCBP2, consistent with its restriction to a more globular conformation. Interestingly, this analysis revealed that the shape of the PCBP2–ΔKH3/SLIVm complex does not differ significantly from that of the PCBP2–FL/SLIVm complex.

Models were generated *ab initio* for the PCBP2-FL/SLIVm complex and for the SLIVm alone. The approximated surface of the PCBP2/SLIVm complex shows similarity to the overall shape and dimensions to that determined using cryoEM, with a globular form and dimensions consistent with the cryoEM-derived models (Figure 2C). Likewise, the derived surface shape for the SLIVm alone bears striking similarity to the cryoEM class representing RNA alone (Figure 2D). These SAXS data thus provide further confirmation of the structures adopted by SLIVm in the presence and absence of PCBP2-FL as they exist in the solution state.

The SAXS data were further interrogated for the information that can be imparted on molecular flexibility. Kratky analysis revealed that an ordering of the protein and RNA structures takes place upon complex formation (Figure 2E). This is indicated mainly by the degree to which the peak of the Kratky profile corresponds to a value of 1.1 at s.Rg = √3 (84). PCBP2-FL and PCBP2-ΔKH3 in their uncomplexed states show a high degree of disorder, as would be expected for these flexible multi-domain molecules. Likewise, the Kratky plot for SLIVm indicates a high degree of disorder for RNA alone. In contrast, the Kratky curve for the PCBP2-FL/SLIVm complex indicates a much more ordered structure. The PCBP2-ΔKH3/SLIVm complex is also relatively ordered, but to a lesser degree than the complex with PCBP2-FL. This indicates that in the absence of the C-terminal residues (that include KH3 and residues 254–287 of the linker region), there is more disorder in the complex structure. Since this disorder could arise from the presence of the untethered 84 residues of the remaining linker region, the PCBP2-KH1/2/SLIVm construct that possesses no remaining linker region was also analysed in the same way for comparison. The PCBP2-KH1/2/SLIVm construct gave rise to a similarly shaped P(r) plot to the previous constructs, indicative of the formation of a globular complex, not dissimilar to that formed by PCBP2-FL and PCBP2-ΔKH3. The Kratky plot indicated a higher degree of order compared to that for the PCBP2–ΔKH3/SLIVm complex, but not the degree of order observed for the PCBP2-FL/SLIVm complex. Together this analysis reveals that the truncated form of PCBP2 is able to form a structured complex with the SLIVm, but that it is slightly more flexible than that formed by PCBP2-FL.

### Accessibility of PCBP2 linker region

**Figure 3.**
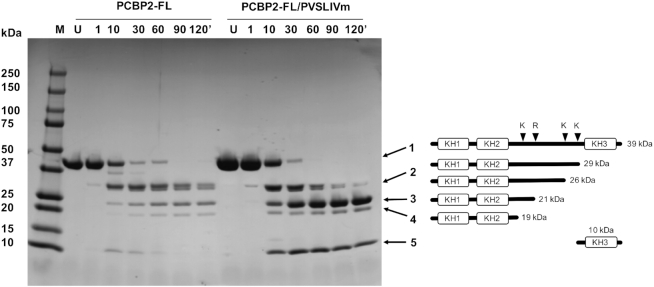
Limited proteolysis of PCBP2-FL free and in complex with SLIVm. Susceptibility of PCBP2-FL to tryptic digest was monitored over time using SDS-PAGE (LHS). M = molecular mass standards; U = untreated protein; 1-120′ denotes the number of minutes of digestion by trypsin. The main bands were identified by N-terminal sequencing. Bands 1 to 4 were all identified as N-terminal fragments and band 5 was identified as KH3. The schematic (RHS) depicts the positions of lysine and arginine residues in the linker region of the full-length PCBP2. Also shown are representations of forms of PCBP2 cleaved at these four sites and their expected molecular masses. These correlate with the apparent masses of the tryptic digest cleavage products in the SDS-PAGE gel. These same bands were seen both in the absence and presence of bound SLIVm. Together this indicates that the PCBP2 linker region is not protected from proteases when PCBP2 is bound to SLIVm.

Neither the cryo-EM nor SAXS experiments revealed the location of the 120 residue linker region of PCBP2. It was thus speculated that this region remains flexible, rather than adopting a compact structure upon forming a complex with SLIVm. We therefore examined the susceptibility of PCBP2-FL to tryptic digestion in the presence and absence of SLIVm. PCBP2-FL and PCBP2-FL/SLIVm were subjected to limited proteolysis over the course of two hours and protein analysed using SDS-PAGE (Figure 3). Main bands were identified using N-terminal sequencing. We observed a digest pattern showing cleavage in the linker region when PCBP2-FL was digested in the absence of SLIVm. There were equivalent levels of cleavage at these sites when PCBP2 was in complex with SLIVm compared to PCBP2 alone. This lack of protection is consistent with the PCBP2 linker region being predominantly unstructured and accessible in the PCBP2-FL/SLIVm complex. Interestingly, KH3 remained visible in the PCBP2-FL/SLIVm sample even after 120 min, suggesting a level of protection through being bound to SLIVm.

### SHAPE and hydroxyl radical cleavage protection analysis of SLIVm

**Figure 4.**
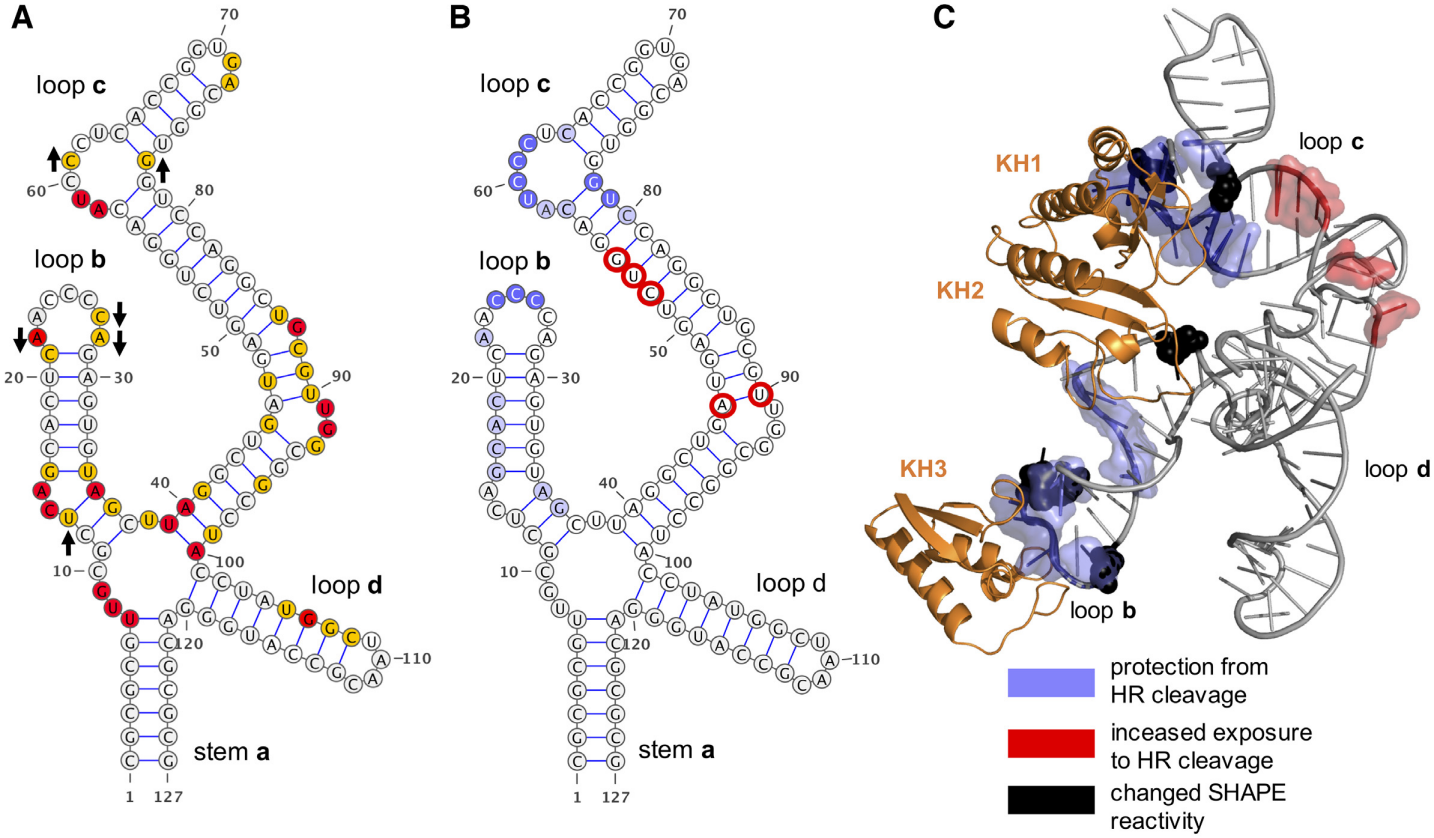
Effect of PCBP-FL binding to SLIVm on SHAPE reactivity and hydroxyl-radical cleavage. (**A**) Schematic of SLIVm RNA topology highlighting SHAPE reactivity of the RNA. Red = Bases with the highest reactivity; Yellow = bases with high reactivity. Arrows denote increased or decreased SHAPE reactivity effected by binding of PCBP2-FL. (**B**) Schematic of SLIVm RNA topology highlighting effect of PCBP2-FL binding on base susceptibility to hydroxyl-radical cleavage. Dark purple = highly protected bases; Light purple = protected bases; Red = decreased protection upon PCBP2-FL binding. (**C**) Mapping of PCBP2-FL binding effects on SLIVm SHAPE reactivity and susceptibility to hydroxyl-radical onto derived model of the PCBP2-FL/SLIVm model.

We performed SHAPE (Selective 2′-Hydroxyl Acylation analysed by Primer Extension) and hydroxyl radical cleavage experiments to determine the effect of PCBP2-FL (and PCBP2-ΔKH3) binding on the SLIVm RNA flexibility and solvent accessibility at individual nucleotides. For this analysis, the SLIVm RNA sequence was cloned into a cassette incorporating 5′ and 3′ additional sequences for primer extension. The SHAPE method utilized NMIA that reacts with ribose 2′-hydroxyl groups that are flexible in the RNA structure, resulting in adduct formation at specific nucleotides. The RNA adduct formation sites were identified using primer extension followed by capillary electrophoresis and the analysed data are shown in Supplementary Figure S4. The SHAPE data plotted onto a 2D representation of SLIVm RNA are shown in Figure 4A. The pattern of reactivity along the nucleotide sequence is consistent with what has been previously reported for SLIV in the context of the whole poliovirus IRES (11), confirming that SLIVm represents an independently and correctly folded domain. Interestingly, the C-rich region at the end of loop **b** of SLIVm is unexpectedly unreactive (as also observed in the context of the whole poliovirus IRES), suggesting an intrinsically structured region. Reactivity was observed in the double stranded region of loop **d** (not observed in the whole poliovirus IRES), suggesting that, in the context of our isolated SLIVm construct, loop **d** has greater flexibility than it does in the entire IRES. Upon repeating the experiment with SLIVm in the presence of bound PCBP2-FL, the SHAPE reactivity pattern remained much the same, as would be expected since RNA reactivity is not known to be sensitive to protein binding. Nevertheless, a few differences were observed in regions of loops **b** and **c** (indicated with arrows). This is consistent with the protein engaging with both of these domains of the RNA and impacting the SHAPE reactivity. These differences were not observed for SLIVm in the presence of PCBP2-ΔKH3, indicating that this truncated protein does not have the same impact on SLIVm structure as PCBP2-FL.

We next implemented RNA footprinting by hydroxyl radical cleavage, a sensitive method to detect RNA sites protected by protein binding. The accessibility to cleavage at each nucleotide was measured in the absence and presence of PCBP2 (see Supplementary Figure S5 for full quantitative data). The data shown in Figure 4B highlight the SLIVm nucleotides that were less accessible to cleavage in the presence of the bound PCBP2. The C-rich regions within loops **b** and **c** were clearly protected, consistent with direct binding by KH1 and KH3 expected at these sites. Adjacent regions of these loops were also protected, although to a lesser degree. Mapped onto the PCBP2/SLIVm model, these regions of protection are clustered together and potentially indicative of a surface that is protected by the, as yet, unlocated linker region. Several nucleotides became more susceptible to cleavage in the presence of PCBP2. These were in the arched region of stem loop **c**, indicative of a structural change induced by the protein binding (Figure 4C).

**Figure 5.**
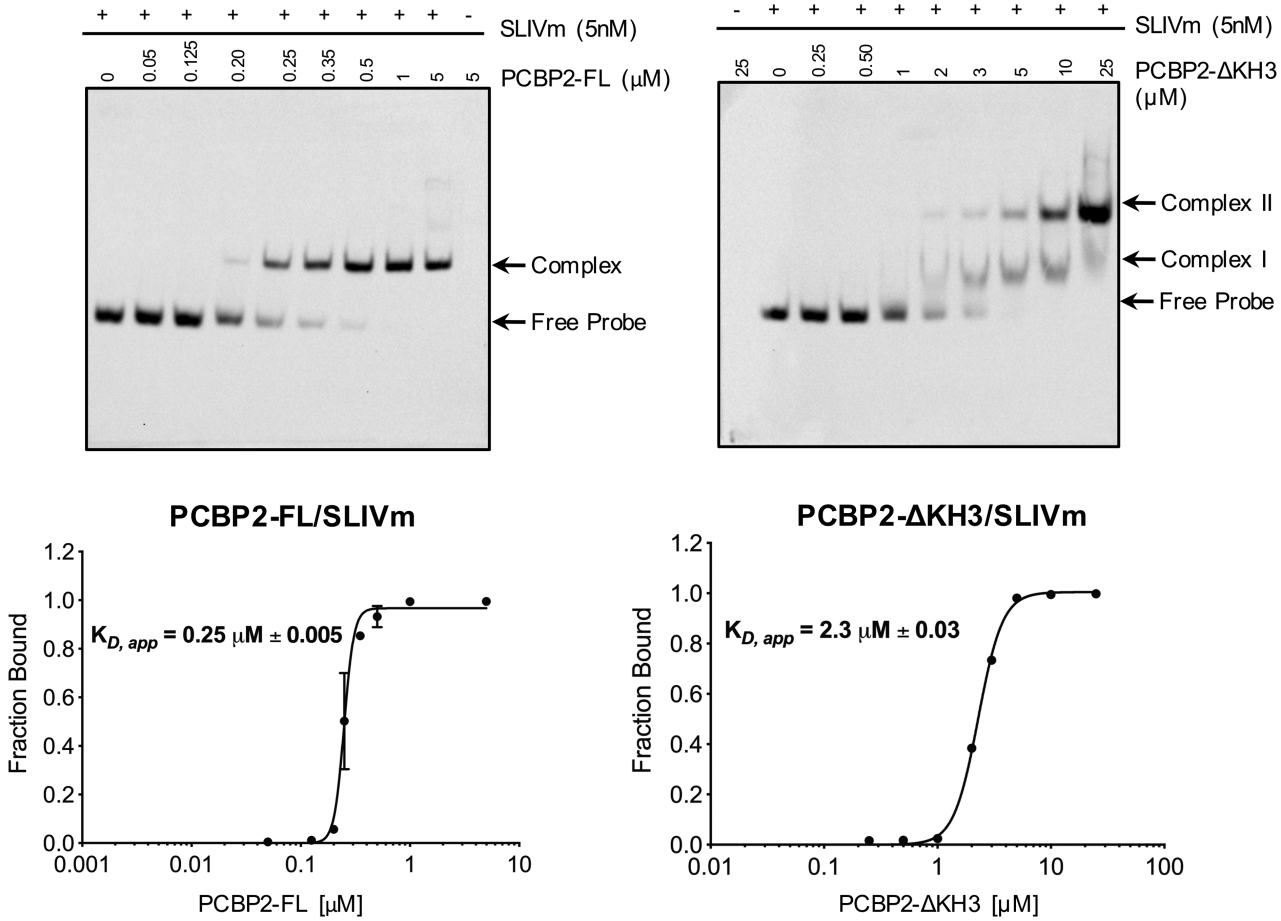
REMSA measurement of PCBP2 construct affinity for SLIVm. Biotinylated SLIVm was tracked in native PAGE after incubation with increasing amounts of PCBP2-FL (LHS) or PCBP2-ΔKH3 (RHS) to compare their binding affinities. *K*_D,app_ values were determined as the protein concentration to effect half maximal binding to the RNA. A higher affinity of *K*_D,app_ = 0.25 μM was observed for PCBP2 and a lower affinity of *K*_D,app_ = 2.3 μM was observed for PCBP2-ΔKH3.

RNA footprinting was also carried out in the presence of PCBP2-ΔKH3 to further investigate the impact of PCBP2 cleavage on the PCBP2/SLIVm structure. No significant difference in cleavage pattern was observed compared to the cleavage of RNA alone, indicating no protection by the presence of this protein. Combined with the lack of impact of PCBP2-ΔKH3 on the SLIVm SHAPE reactivity, this likely reflects a lower stability of complex formation by PCBP2-ΔKH3 compared with PCBP2-FL. To quantitate the stability of complexes formed by PCBP2-ΔKH3 versus PCBP2-FL with SLIVm, the binding affinities were determined using RNA electrophoretic mobility shift assay (REMSA; Figure 5). This confirmed a 10-fold lower binding affinity of PCBP2-ΔKH3 (*K*_D,app_ = 2.3 μM) compared to PCBP2-FL (*K*_D,app_ = 0.25 μM) for SLIVm. Interestingly, while a single shift was observed for PCBP2-FL, two binding transitions were observed for PCBP2-ΔKH3 suggesting a 2:1 protein:RNA stoichiometry for this construct at high protein concentrations. From these findings, it is likely that the PCBP2-ΔKH3 protein concentrations of approximately 0.75 μM utilized for SHAPE and footprinting analyses were insufficient to effect protection of the RNA.

### Impact of PCBP2 cleavage on binding to full-length SLIV

Our structural and binding data suggest a molecular mechanism by which translation of the poliovirus genome is ceased upon PCBP2 cleavage. It does not appear to be via a change to the structure of the apex region of SLIV, since SEC-SAXS showed that the PCBP2-ΔKH3/SLIVm complex retained much the same structure as the PCBP2-FL/SLIVm complex, albeit with greater flexibility. Rather, IRES loss of function may be due to the loss of affinity of PCBP2 upon cleavage. The binding data showed that the affinity of PCBP2-ΔKH3 for SLIVm is diminished compared to PCBP2-FL. This would, over time, result in dissociation of PCBP2 from the IRES rendering it dysfunctional. Our cryoEM data showed that the structure of the unbound SLIVm is that of a flexible RNA cruciform with no resemblance to the structure of the PCBP2/SLIVm complex.

To further explore this possibility, we also tested the binding of PCBP2-FL to the full length SLIV (nt 234–440; SLIV-FL) and compared this to the binding of PCBP2-ΔKH3. We considered that the longer stem of SLIV-FL could contribute to PCBP2-FL binding affinity and potentially also to PCBP2-ΔKH3 binding affinity. RNA electrophoretic mobility shift assays were carried out to compare the binding of both PCBP2-FL and PCBP2-ΔKH3 to SLIV-FL (Supplementary Figure S6). This analysis revealed that PCBP2-FL indeed interacts with higher affinity to the SLIV-FL (*K*_D,app_ = 0.17 μM) compared with SLIVm. Again, however, the affinity is diminished in the case of PCBP2-ΔKH3 (*K*_D,app_ = 0.51 μM). While these experiments do not test the binding of PCBP2 and PCBP2-ΔKH3 in the context of the whole IRES and in the presence of other required eIFs and ITAFs, they are consistent with the loss of IRES function being due to the loss of PCBP2 binding affinity upon cleavage.

## DISCUSSION

PCBP2 binding to the poliovirus IRES SLIV has been shown to be essential for docking of the 40S ribosomal subunit and initiation of translation (20,21). When PCBP2 is cleaved in its linker region by the action of the poliovirus encoded 3CD proteinase, translation ceases and the RNA replication phase of the viral production is able to commence (23,24). The C-rich loop structure of SLIV is also conserved in the type I IRES of other enteroviruses and rhinovirus strains (85), with the requirement for PCBP2 verified for bovine enterovirus (BEV), enterovirus71 (EV71) coxsackie virus B3, cadicivirus and human rhinovirus (HRV) (20,24,56,86). This mechanism is therefore likely to be broadly utilized but has been most rigorously studied in the poliovirus model system. The PCBP2/SLIV structure occurs adjacent to stem–loop V (SLV), that recruits eIF4G to make the primary interaction with eIF3 on the 40S ribosomal subunit. It is therefore speculated that somehow the PCBP2/SLIV provides an extra interaction surface, alongside eIF4G/SLV, for functional assembly of the 43S pre-initiation complex. This has raised key biological questions as to the 3D structure of the PCBP2/SLIV complex and why, when PCBP2 is cleaved, the IRES is no longer functional.

We report the first three-dimensional model of PCBP2 bound to the apex of SLIV that we have called ‘SLIVm’ based on cryoEM data and supported by SAXS data. The 127-nt SLIVm (encompassing nucleotides 278–398 of the poliovirus IRES) comprises loops **b**, **c** and **d** in a cruciform arrangement with the stem **a**, that numerous previous biochemical and biophysical studies have established as the binding sites for the PCBP2 KH domains (54). In particular, it has previously been shown through elegant directed hydroxyl radical cleavage experiments that KH1 of PCBP2 binds at the C-rich region of loop **c** that is adjacent to a GNRA tetraloop (20,56). This same study placed both PCBP2 KH3 and KH2 close to loop **b** and also, although to a lesser extent, close to loop **d**. Our 6.1 Å cryoEM map revealed a globular shape that allowed the PCBP2 KH domains and cruciform shaped SLIVm to be built in a conformational arrangement consistent with these known interactions. There were two classes that we could distinguish that differed in the angle of the coaxially stacked stem **a** and loop **d** at the four-way junction. This may reflect flexibility or conformational alternatives of this structure at the apex of SLIV, or the structure may be stabilized in the presence of the rest of SLIV and neighbouring stem–loops of the IRES.

The most compelling feature of the PCBP2-FL/SLIVm structure is the GNRA tetraloop that protrudes from the ‘fist’ of the KH1/KH2 back-to-back dimer. The importance of this tetraloop was also suggested in a study of the analogous stem–loop of cadicivirus (named stem loop 10) (56). The role of the GNRA tetraloop is not known. GNRA tetraloops are recognized for both their stabilizing effects on RNA structure and for their ability to make interactions with target RNA structures such as minor grooves at the site of two consecutive purines (87). As suggested by the current structure, it may either be that the GNRA tetraloop confers stability to loop **c** to enhance PCBP2 binding or that PCBP2 binding presents the GNRA tetraloop in an optimal way for making distal interactions. Data presented in Supplementary Figure S7 suggest that the latter is more likely. Mutations made to the GNRA tetraloop of poliovirus SLIV do not dramatically compromise PCBP2 binding, but do destroy the functionality of the IRES, both in terms of translation and virus infectivity. The cryoEM density shows the GNRA tetraloop to be a very well defined and rigid feature of the PCBP2/SLIV complex and well positioned to make interactions with neighbouring regions of the IRES or with the surface of the ribosome itself.

The cryoEM-modelled structure clearly shows that the PCBP2/SLIVm complex forms a globular shape with the long loop **c** constrained by PCBP2 towards loop **b**. This is in contrast to the cryoEM derived shape of the SLIVm RNA alone, in which only the junction can be seen and not the end of the longest loop, likely due to flexibility. This is corroborated by the SAXS data that also show a globular form for the PCBP2/SLIVm complex and an elongated *ab initio* shape for the SLIVm alone. Thus, PCBP2 binding of the C-rich loop **c** via KH1 appears to induce an arched dsRNA structure that brings the other KH domains in close proximity to loop **b**. In the current model KH3 is positioned at the C-rich region of loop **b**, since KH3 is able to interact with this motif with good affinity and the structural basis for this interaction is also known (30,32). KH2, in the model, is positioned close to the stem of loop **b** and could make an interaction that tethers PCBP2 to the RNA in this vicinity. It is known that KH2 is capable of binding to C-rich RNA (34) and contributes to affinity for SLIV (35), but the affinity of KH2 has not been separately measured nor specificity of binding characterized. There is no third C-triplet sequence in SLIVm, however, that is an obvious target site for KH2; so it will remain for further studies to position this domain accurately.

The linker between KH2 and KH3 in PCBP2 could not be observed in the cryoEM model, suggesting that the linker is flexible - at least in part. There is a portion of unexplained cryoEM map density that could represent an ordered region of the linker region positioned against the SLIVm surface, but the majority of the linker is likely to be mobile. This is consistent with the tryptic digest results that showed a series of cleavages along the PCBP2 linker region that were not protected by interaction with SLIVm. The linker region thus remains somewhat enigmatic. On one hand it is this linker region that differentiates PCBP2 from PCBP1, that is unable to support poliovirus IRES function (55). This suggests that a specific interaction of the PCBP2 linker region with the SLIVm takes place. On the other hand, the linker region must be accessible for cleavage by 3CD in the process of viral replication. In this case, its flexible nature is consistent with its function.

Additional support for our overall structural model is provided by SHAPE and hydroxyl radical cleavage protection studies. SHAPE confirmed the topological arrangement of the SLIVm out of the context of the rest of the IRES, although it suggested some extra flexibility in loop **d**. The regions of SLIVm that were relatively protected from hydroxyl radical cleavage by PCBP2 binding were consistent with the model – being mainly positioned at the KH domain binding sites and also at the site of putative linker interaction. SLIVm SHAPE and hydroxyl radical cleavage protection studies, however, showed no difference in the presence of PCBP2-ΔKH3, even at loop **c** where KH1 would still bind. Even though the SAXS data showed the PCBP2-ΔKH3/SLIVm complex to adopt a compact globular structure not dissimilar from the PCBP-FL/SLIVm structure, we determined its affinity to be 10-fold lower. Thus, under the conditions of the SHAPE and hydroxyl radical cleavage experiments, PCBP2-ΔKH3 was likely insufficiently bound to SLIVm to have an impact on SHAPE reactivity or susceptibility to hydroxyl radical cleavage.

The difference between the PCBP2-FL and PCBP2-ΔKH3 affinity for SLIVm prompted us to consider whether this differential affinity would also be seen in the context of a larger portion of SLIV. Both proteins bound with higher affinity to a full-length SLIV construct compared to SLIVm, but PCBP2-FL binding remained ∼3-fold higher affinity compared to that of PCBP2-ΔKH3. In the context of the intact IRES in the cell, the interactions may be higher affinity as well, but it is likely that PCBP2-FL will maintain a greater affinity than PCBP2-ΔKH3. With an approximated cellular concentration of PCBP2 of 100 nM (88,89), this may support complex formation of PCBP2-FL but not PCBP2-ΔKH3.

Taken together, our data support a model of molecular switching that occurs upon cleavage of PCBP2 by 3CD due to the reduced affinity of PCBP2 for poliovirus SLIV. Even though PCBP2-ΔKH3 is able to maintain the complex structure via KH1 and KH2 binding, we propose that its reduced affinity renders it ineffective for facilitating IRES function. In contrast, the cleaved form of PCBP2 is still sufficient for complex formation with the 5′-cloverleaf structure of the poliovirus genome that facilitates viral replication (23,24). Here it should be noted that the 5′-cloverleaf is also a cruciform RNA structure with a C-rich region in one of the loops that is the target of PCBP2, so superficially may bear some resemblance to the PCBP2/SLIVm structure despite it possessing a completely different function. Structural information for this complex is yet to be elucidated. Thus PCBP2 cleavage results in a dysfunctional IRES and permits the commencement of viral RNA synthesis on the template that has been cleared of translating ribosomes.

**Figure 6.**
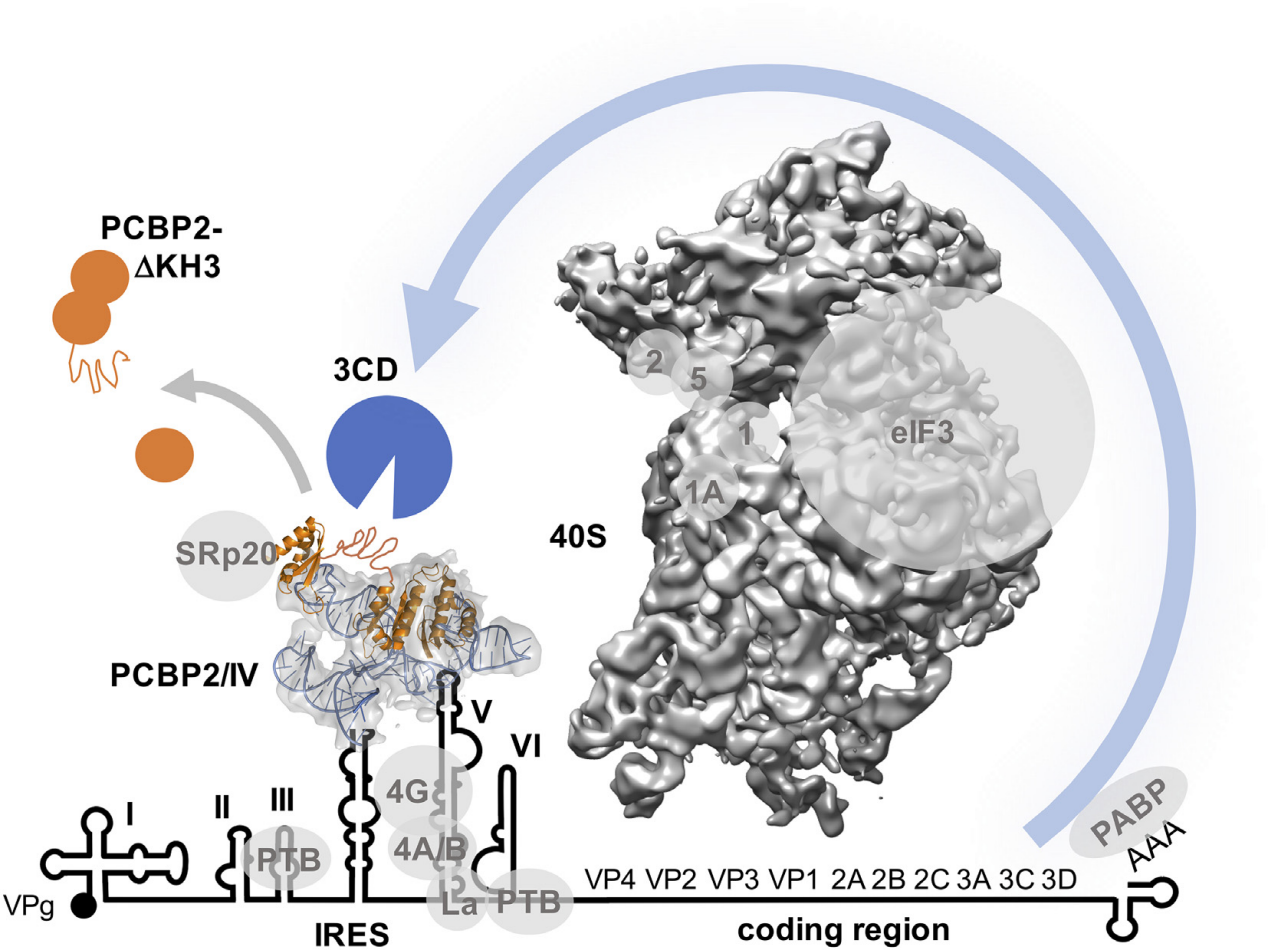
Schematic depiction of the potential role of the PCBP2/SLIV complex. The PCBP2 interaction with stem loop IV occurs at the centre of the poliovirus IRES and adjacent to stem loop V that is the binding site for critical eukaryotic initiation factors eIF4G and 4A/B. PCBP2 may assist in stabilizing a required IRES conformation, or it may augment interactions with the 40S ribosomal subunit through direct interactions or via eIFs and also potentially through interactions with SRp20. Also depicted are other poliovirus ITAFS as well as the virally encoded 5′-attached protein VPg. The linker region is depicted as an accessible unstructured region, that is cleaved by the virally encoded 3CD proteinase resulting in the loss in affinity of PCBP2 for the IRES and consequent loss of IRES function.

Exactly why the full-length PCBP2/SLIV complex is required for IRES function is not yet known. It is possible that KH3 plays a role recruiting other factors (such as SRp20) that support IRES function until KH3 is lost, but since PCBP2 is the only essential factor, the main role of PCBP2 binding to poliovirus SLIV is likely to be structural. The current work thus provides the first picture of this structural arrangement. It suggests that loop **c** is configured very precisely with a protruding GNRA tetraloop. It remains to be determined whether this facilitates further interactions with other regions of the poliovirus IRES or whether it forms direct interactions with the 40S ribosomal subunit alongside eIF4G that is bound to SLV. A schematic summarizing our current understanding is presented in Figure 6. This structure, that is essential for poliovirus translation, suggests that features such as the apex of loop **c** containing the GNRA tetraloop, in particular, may be key to its function. The structural information for the poliovirus PCBP2-SLIV interaction domains reported in this study thus provides a starting point for the development of novel antivirals that disrupt these sites of interaction. Given that other picornaviruses like human rhinovirus, coxsackievirus, and EV71 require the conserved interaction of PCBP2 with the IRES present in the 5′-untranslated regions of their genomic RNAs, our findings also have broad implications for the underlying replication mechanisms used by these viruses.

## DATA AVAILABILITY

Data supporting the findings of this manuscript are available from the corresponding author upon reasonable request. The cryo-EM map describing the PCBP2/Stem Loop IVm class 1 structure is deposited in the Electron Microscopy data Bank (EMDB) under accession code EMD-21133.

SAXS data have been deposited in the Small Angle Scattering Biological Data Bank (SASBDB) under the accession numbers: SASDH55: SLIVm; SASDH65: PCBP2; SASDH75: PCBP2-FL/SLIVm; SASDH85: PCBP2-ΔKH3/SLIVm; SASDH95: PCBP2-ΔKH3; SASDHA5;PCBP2-KH1/2/SLIVm.

## Supplementary Material

gkaa519_Supplemental_File

## Notes

Present address: Simone A. Beckham, La Trobe Rural Health School, College of Science, Health and Engineering, La Trobe University, Victoria 3550, Australia.
