# Supplementary material for: Structure of the PCBP2/stem–loop IV complex underlying translation initiation mediated by the poliovirus type I IRES
Source: Nucleic Acids Res. 2020 Jun 18;48(14):8006–21. doi: 10.1093/nar/gkaa519 (PMC7641305; doi:10.1093/nar/gkaa519)
Supplement: gkaa519_Supplemental_File [file gkaa519_supplemental_file.pdf]

SUPPLEMENTARY DATA

Structure of the PCBP2/Stem Loop IVm complex underlying the functionality of the poliovirus type I IRES.

Simone A Beckham, Mehdi Y Matak, Matthew J Belousoff, Hariprasad Venugopal, Neelam Shah, Naveen Venkadari, Hans Elmlund, Joseph HC Nguyen, Bert L Semler, Matthew CJ Wilce and Jacqueline A Wilce

Supplementary Figure 1. SEC-MALS Chromatograms for PCBP2/SLIVm constructs

Supplementary Figure 2. Cryo-EM data reconstruction of PCBP2-FL/SLIVm and SLIVm

Supplementary Figure 3. Gaussian deconvolution of SEC-SAXS data

Supplementary Figure 4. SHAPE data for SLIVm-SHAPE RNA

Supplementary Figure 5. Hydroxyl-radical (HR) cleavage of SLIVm-SHAPE RNA

Supplementary Figure 6. REMSA measurement of PCBP2 construct affinity for the full-length SLIV (SLIV-FL)

Supplementary Figure 7. Role of the GNRA tetraloop in poliovirus stem-loop IV RNA-protein interactions and translation functions.

Supplementary Table 1. Molecular masses estimated from SEC-MALS

Supplementary Table 2. SAXS derived structural parameters for PCBP2 and SLIVm RNA

Supplementary Figure 1. SEC-MALS Chromatograms for PCBP2/SLIVm constructs

Shown are the size exclusion chromatography elution profiles recorded at 280 nm and 260 nm for PCBP2 and SLIVm constructs before and after complex formation. Simultaneous multi-angle light scattering (MALS) estimation of molecular mass is also shown as a scatter plot. Profiles are superposed to show that the PCBP2/SLIVm complexes form in a 1:1 ratio, elute at an earlier time and can be prepared in pure form. **A)** Superposed profiles of PCBP2-FL, SLIVm and PCBP2-FL/SLIVm at 280 nm (---) and 260 nm (-); **B)** Superposed profiles of SLIVm and PCBP2-KH/1/2/SLIVm at 280 nm (---) and 260 nm (-); **C)** Superposed profiles of PCBP2-ΔKH3, SLIVm and PCBP2-ΔKH3/SLIVm at 280 nm (---) and 260 nm (-); **D)** Superposed profiles of SLIVm-SHAPE RNA construct (···) and complexes formed with PCBP2-FL (-) or PCBP2-ΔKH3 (---) at both 280 nm and 260 nm.

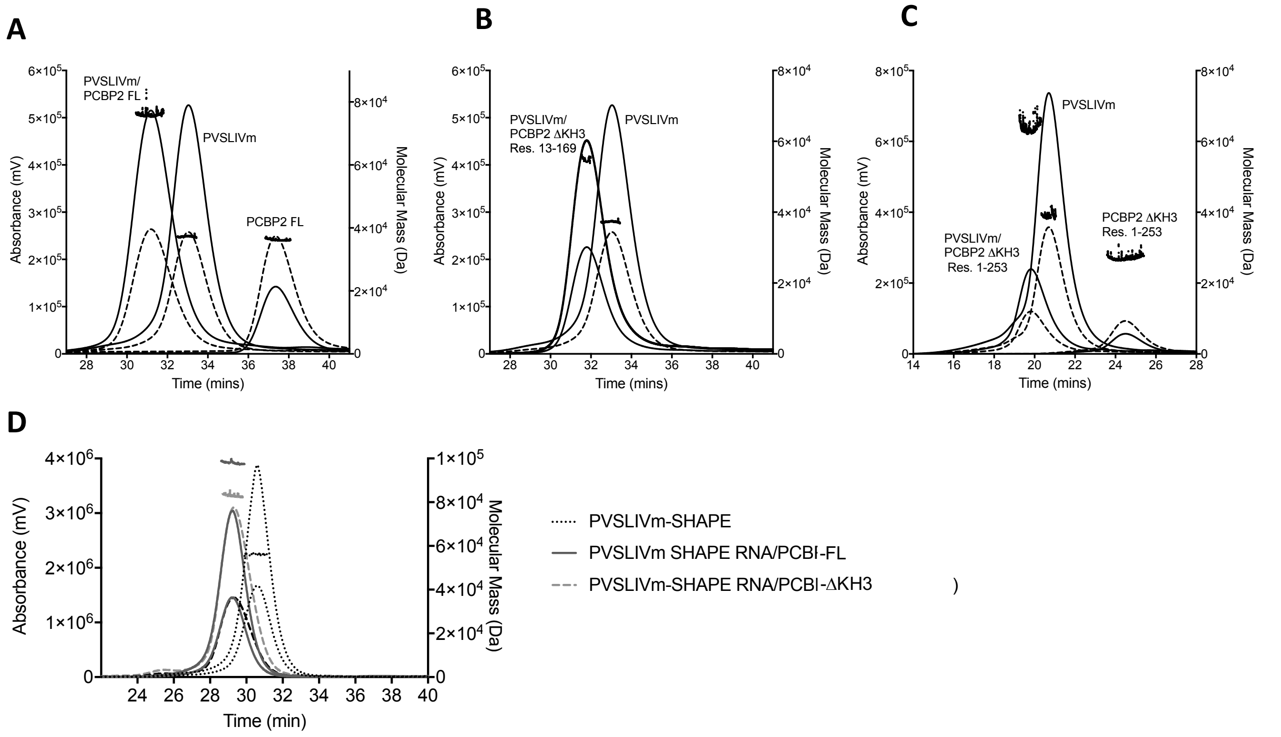

Supplementary Figure 2. Cryo-EM data reconstruction of PCBP2-FL/SLIVm and SLIVm
A) Representative micrograph showing particle distribution of PCBP2-FL/SLIVm (circled in *yellow*) and SLIVm from which 744,895 particles were picked for further data processing; **B)** Representative 2D classes computationally constructed from 265,865 particles; **C)** 3D classification of two reconstructed PCBP2-FL/SLIVm classes and a class representing RNA alone; **D)** Fourier Shell Correlation (FSC) curves for the reconstructed PCBP2-FL/SLIVm classes.

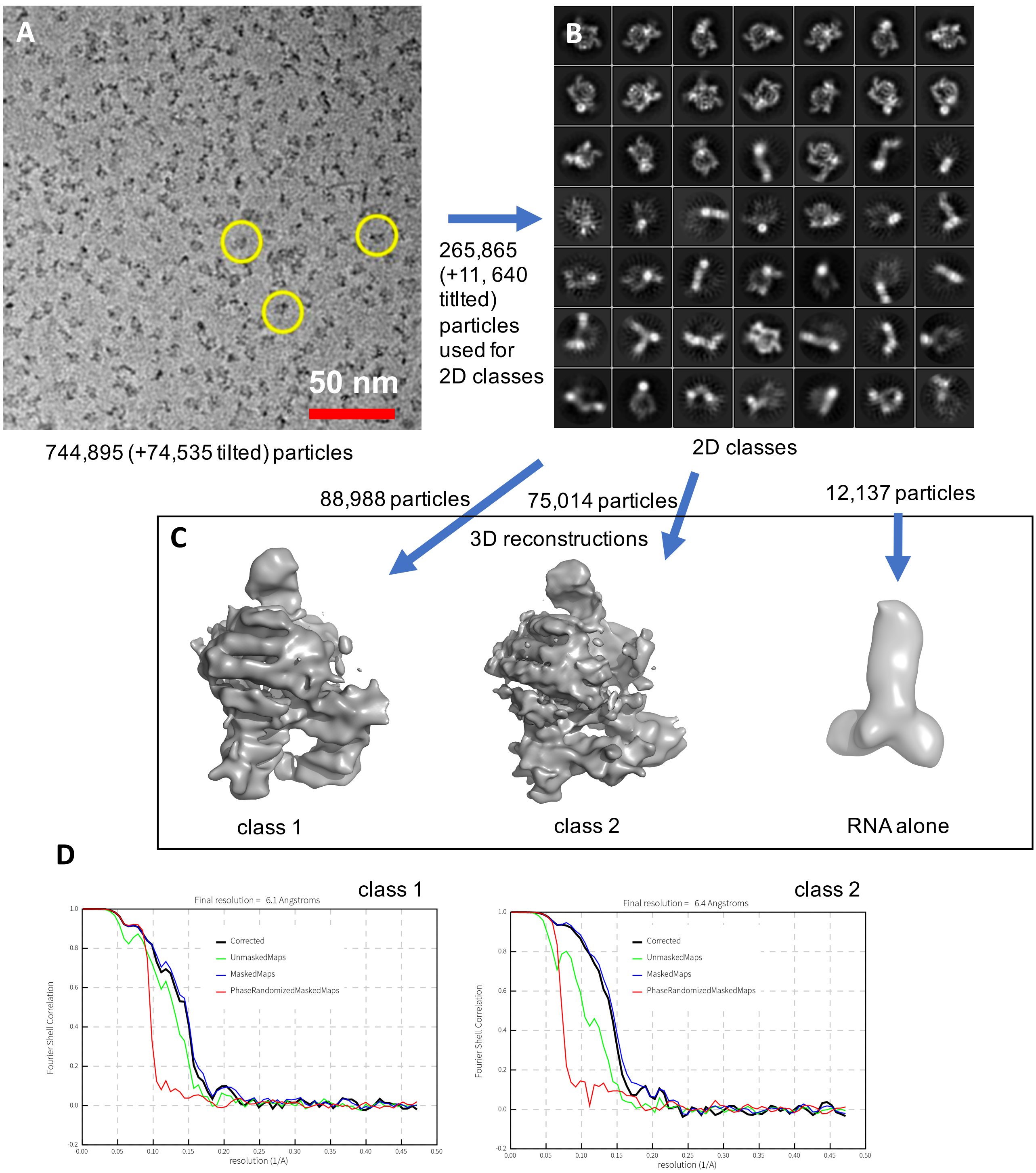

Supplementary Figure 3. Gaussian deconvolution of SEC-SAXS data

Shown are the scattering intensity profiles (grey dots) of PCBP2, SLIVm and PCBP2/SLIVm constructs collected as they eluted from the size exclusion column. Gaussian deconvolution of the peaks (named Pk1, Pk2, Pk3) was conducted in order to deconvolute scattering data of the main species (red curves) from earlier eluting species (green or blue curves). Also shown are the Rg values calculated for each of the deconvoluted peaks (coloured dots).

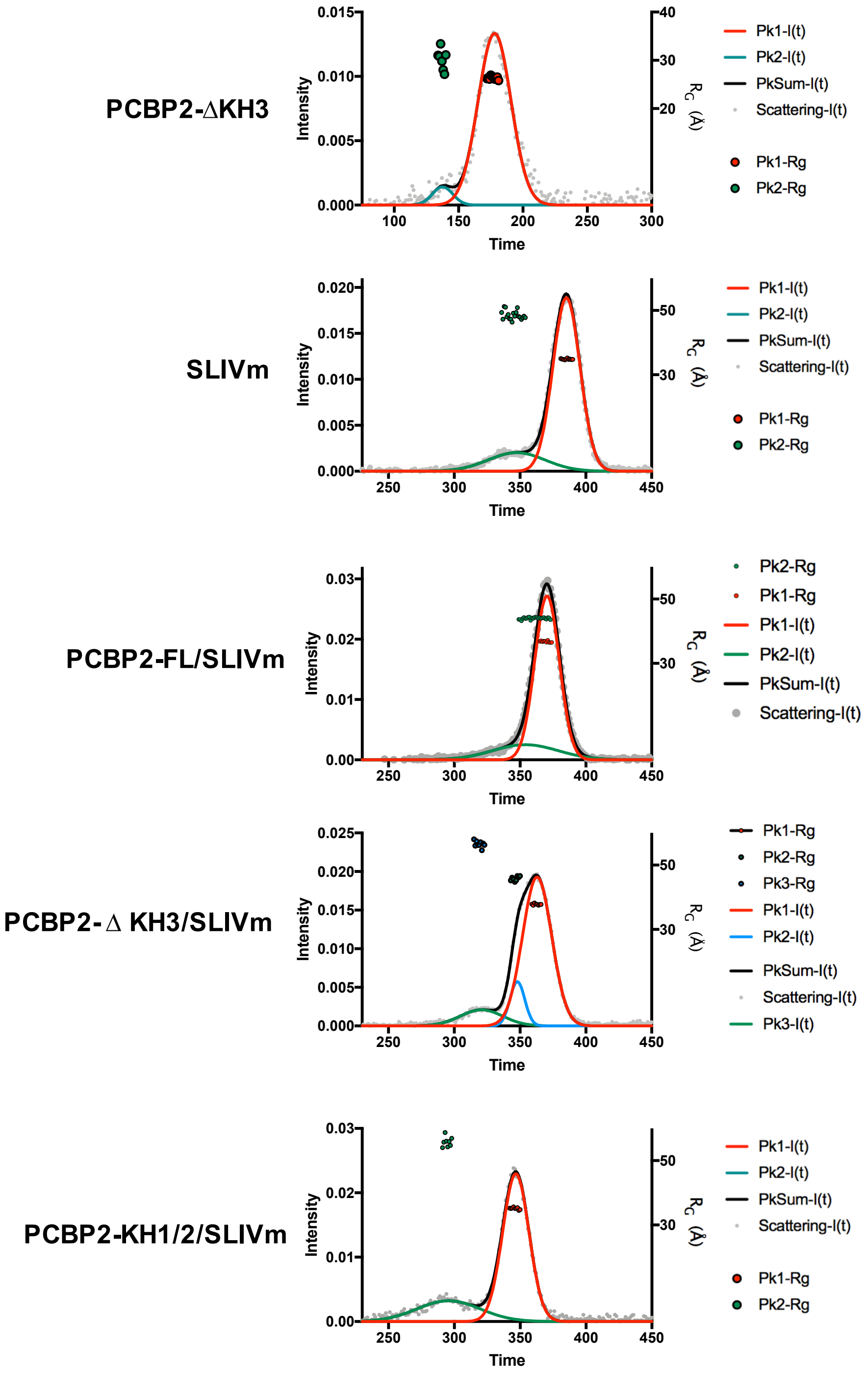

Supplementary Figure 4. Selective 2'-hydroxyl acylation analysed by primer extension (SHAPE) data. The SHAPE analyses are shown for SLIVm-SHAPE RNA including nucleotides 279-395 of the poliovirus genome (calculated as described in the Materials and Methods section). The blue bars represent the SHAPE reactivity of individual nucleotides within the SLIVm-SHAPE RNA alone. Values above an arbitrary value of 0.5 are designated with a red dot (highly reactive), and those above 0.25 with a yellow dot (reactive). The grey bars represent the SHAPE reactivity of SLIVm-SHAPE RNA nucleotides in the presence of PCBP2-FL. Increases or decreases in reactivity are indicated by up arrows. The orange bars represent the SHAPE reactivity of SLIVm-SHAPE RNA nucleotides in the presence of PCBP2-ΔKH3.

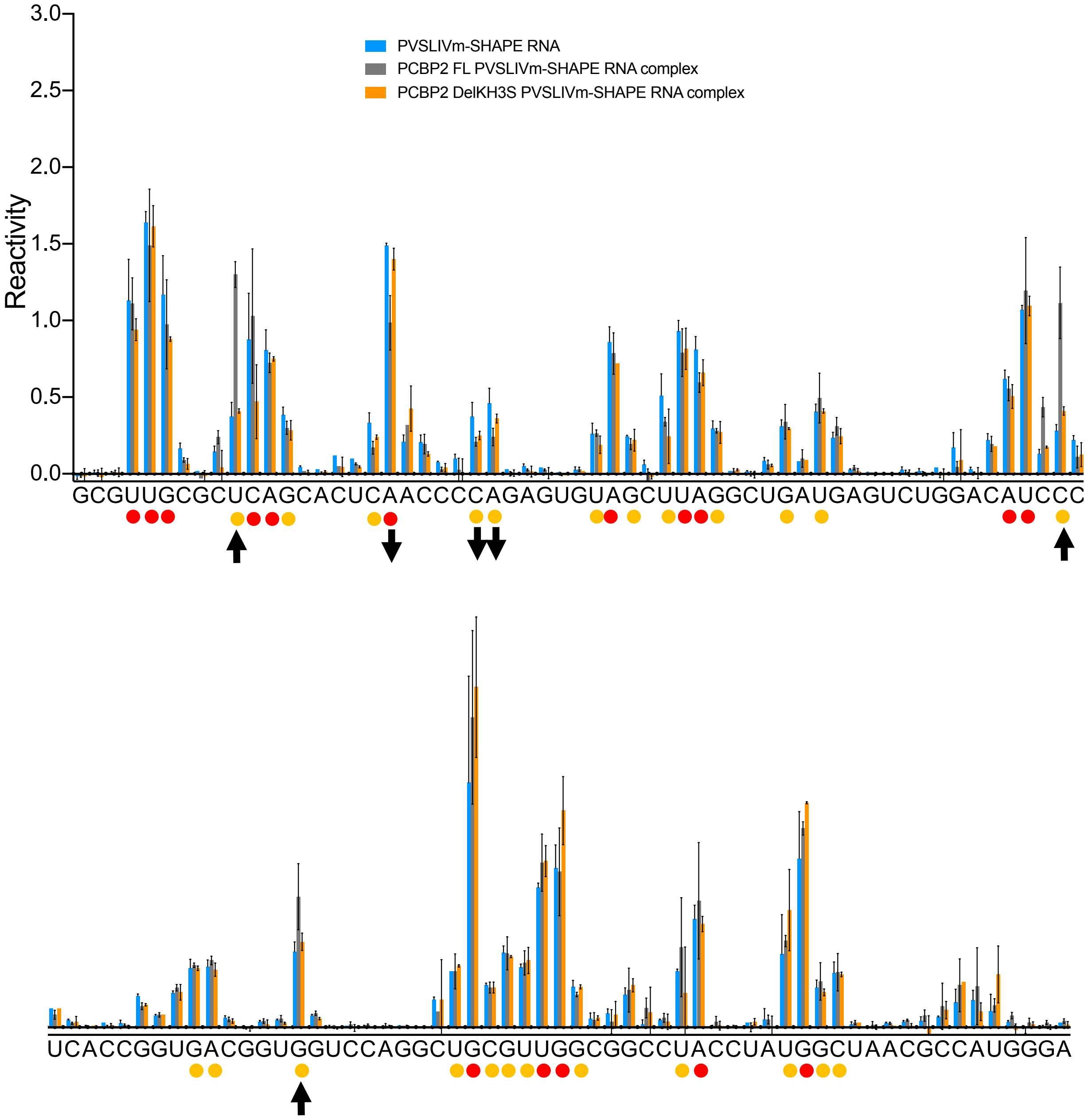

Supplementary Figure 5. Hydroxyl-radical (HR) cleavage of SLIVm-SHAPE RNA. HR cleavage data are shown for SLIVm-SHAPE RNA including nucleotides 279-395 of the poliovirus genome (calculated as described in the Materials and Methods section). The blue bars represent the HR reactivity of individual nucleotides within the SLIVm-SHAPE RNA alone. The grey bars represent the SHAPE reactivity of SLIVm-SHAPE RNA nucleotides in the presence of PCBP2-FL and the orange bars represent the SHAPE reactivity of SLIVm-SHAPE RNA nucleotides in the presence of PCBP2-ΔKH3. Significant differences were only effected by PCBP2-FL. These nucleotides are highlighted with pale purple dots (decrease in HR cleavage) or dark purple dots (large decrease in HR cleavage) or red circle (increase in HR cleavage).

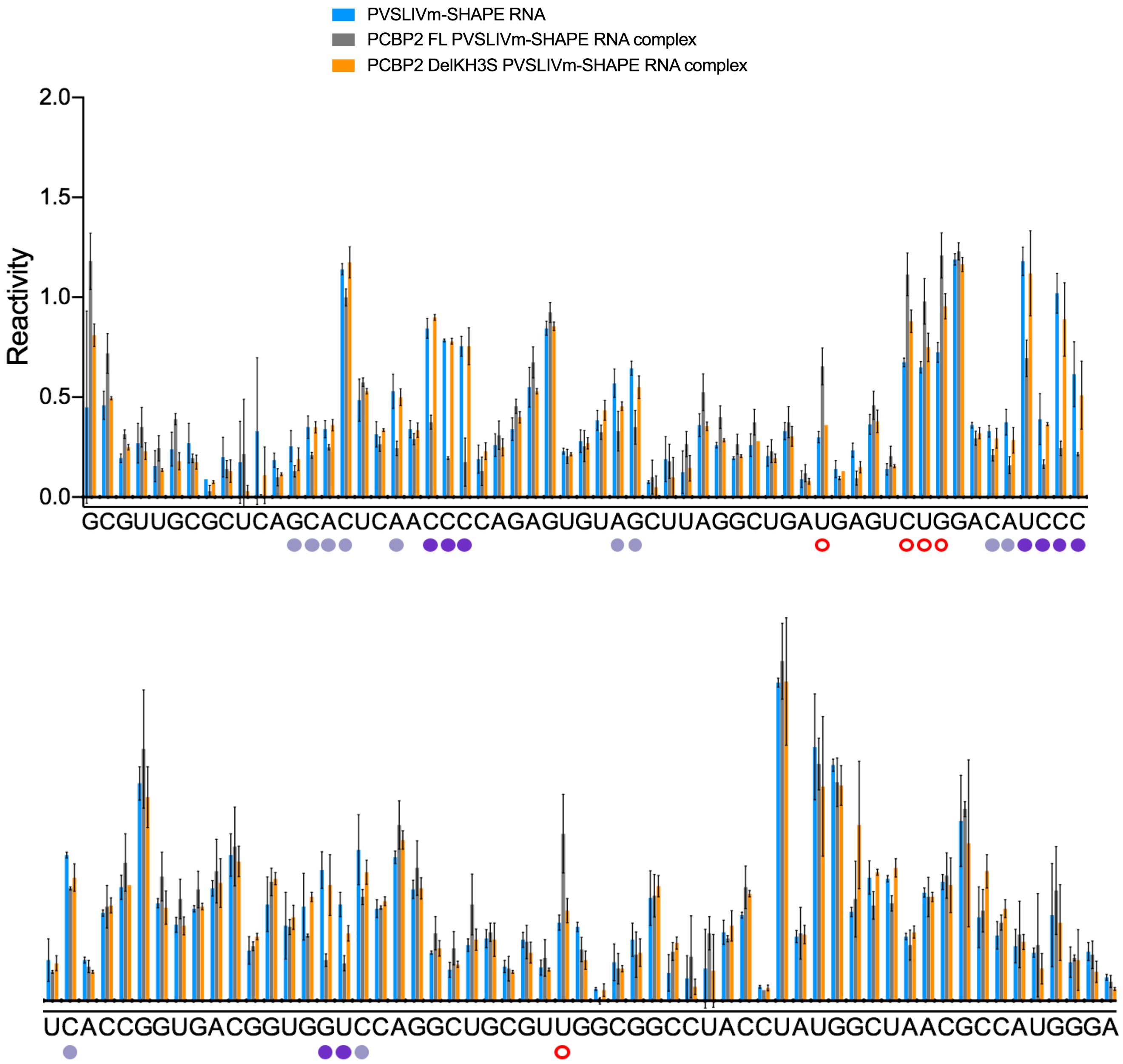

Supplementary Figure 6. REMSA measurement of PCBP2 construct affinity for the full-length SLIV (SLIV-FL). SLIV-FL RNA (nt 234-440) was prepared in the same way as for SLIVm (described in the main text) but using a DNA template amplified from the pT220-460 IRES plasmid using a forward oligonucleotide directed against nucleotides 234-254 and encoding the T7 RNA polymerase promotor site at the 5'-terminus (5' TAATACGACTCACTATAGGGCTTATGTACTTCGAGAAGCCC-3'), and a reverse oligonucleotide designed against nucleotides 417-440 (5'-GCTTATGTAGCTCAATAGGCTCTTCA-3'). The REMSA was run as also described in the main text. Biotinylated SLIV-FL was tracked in native PAGE after incubation with increasing amounts of PCBP2-FL (LHS) or PCBP2-ΔKH3 (RHS) to compare their binding affinities. $K_{D,app}$ values were determined as the protein concentration to effect half maximal binding to the RNA. A higher affinity of $K_{D,app}$ = 0.17 µM was observed for PCBP2 and a lower affinity of $K_{D,app}$ = 0.51 µM was observed for PCBP2-ΔKH3.

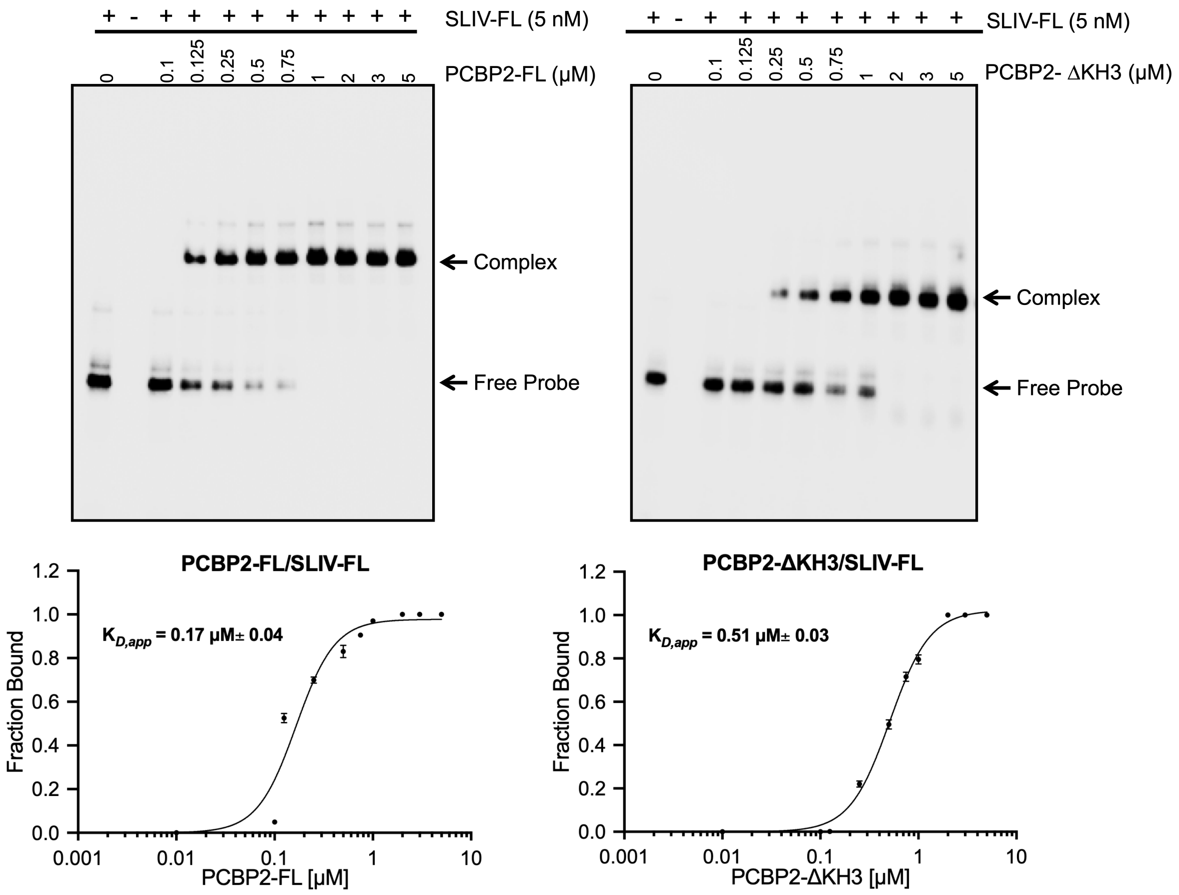

Supplementary Figure 7. Role of the GNRA tetraloop in poliovirus stem-loop IV RNA-protein interactions and translation functions. A) Predicted RNA secondary structure of the upper portion of poliovirus stem loop IV (SLIV) RNA, showing specific nucleotide numbers. The half-circled nucleotides represent the GNRA tetraloop sequences (GUGA) and the mutated version of those sequences (GACG). **B)** RNA electrophoretic mobility shift analysis with ^{32}P-labeled SLIV RNA (nucleotides 220-460) from the poliovirus 5' noncoding region of the genome, either wild type (WT) or containing the GNRA substitution mutation (GNRA-Sub). Increasing amounts of recombinant full-length poly(C) binding protein 2 (PCBP2-FL) were incubated with the labeled RNA probe (approx. 0.1 nM) as previously described (1). The resulting RNP complexes (denoted in the figure) were resolved at 4°C on a native 4% polyacrylamide gel. **C)** Poliovirus *in vitro* translation reactions. Full-length, wild type (WT) or mutated (GNRA-Sub) genomic RNAs were generated by *in vitro* transcription of plasmid pT7PV1, which harbors an infectious cDNA copy of the poliovirus genome. Translation reactions were carried out in S10 extracts from uninfected HeLa cells in the presence of ^{35}S methionine as previously described (1). Incubation times were either 2 hr or 4 hr at 30 °C with either 150 ng or 300 ng of transcript RNA in a total volume of 10 µl. Following incubation, Laemmli sample buffer was added to each reaction and the resulting mixture was subjected to electrophoresis on an SDS-containing, 12.5% polyacrylamide gel. The gel was subjected to fluorography and subsequent autoradiography. The (M) lane corresponds to a marker lysate derived from poliovirus-infected HeLa cells incubated in the presence of ^{35}S-methionine, with the arrows denoting bands corresponding to specific poliovirus proteins. The (-) lane corresponds to an incubation that was carried out in the absence of viral RNA. **Ref 1**: Walter et. Al., (2002) *J Virol* **76**, 12008-12022.

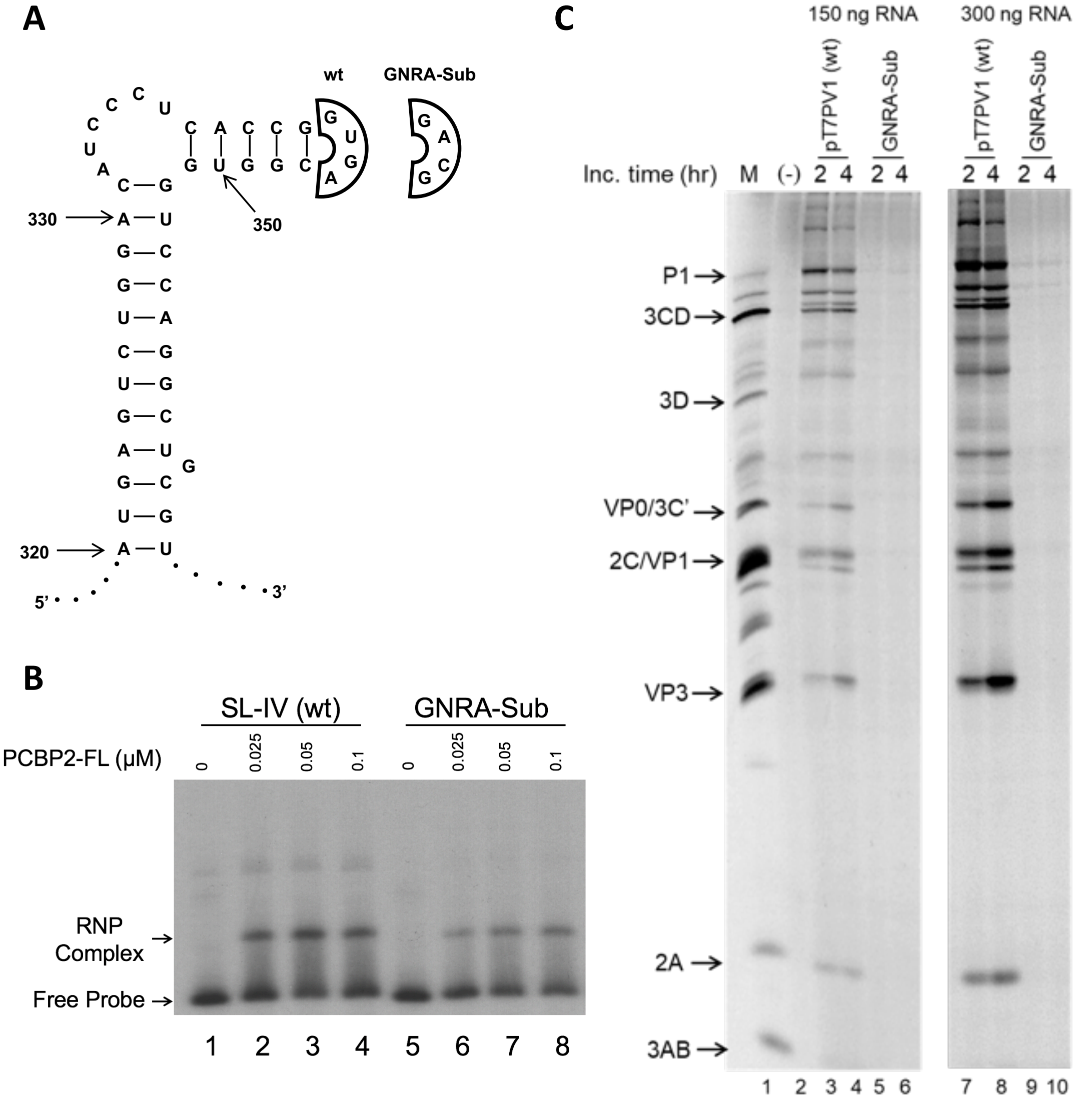

Supplementary Table 1
Molecular masses estimated from SEC-MALS

	Predicted MW	MALS MW
PVSLIVm	41,211	40,580 (+/- 0.67%)
PCBP2-FL	39,645	38,020 (+/- 0.39%)
PCBP2-ΔKH3(1-253)	27,969	24,530 (+/- 1.05%)
PCBP2-KH1/2 (13-169)	19,176	14,570 (+/- 0.80%)
PCBP2-FL/SLIVm	80,856	77,800 (+/- 0.54%)
PCBP2-ΔKH3/SLIVm	69,180	68,940 (+/- 3.09%)
PCBP2-KH1/2/SLIVm	60,387	51,280 (+/- 0.33%)
PVSLIVm-SHAPE RNA	58,834	56,040 (+/- 0.88%)
PVSLIVm-SHAPE RNA-PCBP2-FL	98,479	98,980 (+/- 0.67%)
PVSLIVm-SHAPE RNA-PCBP2-ΔKH3(1-253)	86,803	82,960 (+/- 0.59%)

Supplementary Table 2
SAXS derived structural parameters for PCBP2 construct and SLIVm RNA samples.

**Sample**	**R_G (Guinier) (Å)**	**I_0 (Guinier)**	**R_G (GNOM) (Å)**	**R_{max} (Å)**	**Calculated MW (kDa)**	**MW from SAXS* (kDa)**
**PCBP2-FL**	33.2 ± 0.3	0.012 $\pm 3.8\times10^{-5}$	34.0	132	39.6	39.1
**PCBP2-ΔKH3**	26.4 ± 0.13	0.011 $\pm 2\times10^{-5}$	26.4	93	28.8	33.8
**SLIVm**	35.0 ± 0.17	0.017 2×10^{-5}	35.8	118	39.2	45.7
**PCBP2-FL/SLIVm**	36.7 ± 0.13	0.027 $\pm 2.5\times10^{-5}$	37.0	115	78.8	85.5
**PCBP2-ΔKH3/ SLIVm**	37.6 ± 0.2	0.021 $\pm 4\times10^{-5}$	38.3	122	68.0	72.4
**PCBP2KH1/2/ SLIVm**	35.01 ± 0.23	0.021 $\pm 4.5\times10^{-5}$	35.6	122	60.4	63.8

* calculated by Bayesian Inference according to: Hajizadeh, N.R., Franke, D., Jeffries, C.M. *&* Svergun, D.I. (2018) Consensus Bayesian assessment of protein molecular mass from solution X-ray scattering data. *Sci Rep* **8,** 7204.
